# Expression of combinatorial immunoglobulins in macrophages in the tumor microenvironment

**DOI:** 10.1371/journal.pone.0204108

**Published:** 2018-09-21

**Authors:** Tina Fuchs, Martin Hahn, Lukas Ries, Sophie Giesler, Svenja Busch, Chunlin Wang, Jian Han, Torsten J. Schulze, Kerstin Puellmann, Alexander W. Beham, Wolfgang E. Kaminski, Michael Neumaier

**Affiliations:** 1 Institute for Clinical Chemistry, Medical Faculty Mannheim, University of Heidelberg, Mannheim, Germany; 2 iRepertoire inc. Huntsville, AL, United States of America; 3 HudsonAlpha Institute for Biotechnology, Huntsville, AL, United States of America; 4 Institute of Transfusion Medicine and Immunology, Medical Faculty Mannheim, University of Heidelberg, Mannheim, Germany; 5 Aesculabor Hamburg, Hamburg, Germany; 6 Department of Surgery, University of Göttingen, Göttingen, Germany; 7 Bioscientia Institute for Medical Diagnostics, Ingelheim, Germany; INSERM, FRANCE

## Abstract

Recent evidence indicates the presence of macrophage subpopulations that express the TCRαβ in chronic inflammatory diseases such as tuberculosis and atherosclerosis and in the tumor microenvironment. Here, we demonstrate that a second subpopulation of macrophages expresses rearranged heavy and light chain immunoglobulins. We identify immunoglobulin expression in human and murine monocytes, in *ex vivo* differentiated macrophages and macrophages from the tumor microenvironment of five randomly selected distinct human tumor entities. The immunoglobulin heavy and light chains are expressed in a small macrophage subfraction (~3–5%) as combinatorial and individual-specific immune receptors. Using Sanger sequencing and deep sequencing, we routinely find markedly restricted Ig repertoires in monocytes/macrophages compared to normal B cells. Furthermore, we report the complete Ig heavy and light chain sequences of a fully functional immunoglobulin from a single tumor-associated macrophage. These results demonstrate that Ig expression is a defining feature of monocytes and also macrophages in the tumor microenvironment and thus reveal an as yet unrecognized modus operandi of host defense in professional phagocytes.

## Introduction

Macrophages are ubiquitous versatile immune cells and key players in major chronic inflammatory diseases [1]. Based on their myeloid origin and their status as professional phagocytes they function as pillars of the innate immune system [2].

Traditional immunological dogma holds that flexible immune recognition in higher vertebrates represented by combinatorial immunoglobulins (Ig) and T cell receptors (TCR) is an exclusive feature of lymphoid effector cells [2,3]. In the past decade, however, a series of studies has provided evidence for the existence of recombinatorial immune receptors outside the lymphoid lineage [4,5]. The initial observation was made in 2006 by Puellmann et al. who demonstrated constitutive expression of variable T cell αβ receptors in a subpopulation of neutrophils [6–8]. Subsequent studies showed production of T cell receptors (TCR) αβ/γδ in monocytes/ macrophages [9,10] and TCRγδ in eosinophils [11]. Importantly, TCR based myeloid variable immune receptors have been implicated in various chronic diseases including autoimmune disease [12], chronic periodontitis[13], tuberculosis [9] and atherosclerosis [14]. Moreover, a most recent study from our laboratory provides evidence for expression of variable TCRαβ by macrophages in the tumor microenvironment [15].

Separate from these studies, recent evidence points to the possibility that myeloid immune effector cells are also capable of expressing immunoglobulin (Ig) heavy and light chain genes [16,17].

In the tumor milieu, cytokines secreted by tumor cells can induce monocyte maturation along an alternative path of activation [18]. These tumor-associated macrophages (TAM) are thought to act as key players in the host’s immune response to tumors. Available evidence suggests roles of TAM in promoting tumor growth and progression [19–22], and marked TAM infiltration has been associated with a poor clinical outcome in tumor patients [23–25]. However, the exact role of macrophages in the tumor microenvironment remains ambiguous.

Given the above observations that two major myeloid cell types i.e. granulocytes and monocytes/macrophages, synthesize combinatorial TCRαβ immunoreceptors and that preliminary evidence suggests Ig expression in monocytes, we tested whether monocytes and macrophages are capable of expressing complete combinatorial immunoglobulins. A particular focus in our study was put on tumor-associated macrophages.

## Materials and methods

### Patient samples

All tumor and blood samples were excess material derived from patients in the course of their medical care. The use of these specimens and mononuclear cells from the healthy donor was approved by the Ethics Committee of the Faculty of Medicine Mannheim, University of Heidelberg (Permit Number: 2014-562N-MA; 2012-293N-MA). All patients provided written informed consent. The samples were strictly anonymized.

### Isolation of macrophages from solid tumors

CD14^+^ cells were isolated from solid tumors. The tumors were finely minced with a scalpel and digested with collagenase IV (190 U/ml) and DNase I (500 U/ml). The cells were washed in cold PBS and erythrocytes were lysed. Cells were further purified using CD14 MACS MicroBeads (Miltenyi Biotec) following the manufacturer’s instructions. For single cell analysis an additional purification step was performed with CD14 Dynabeads (Life technologies).

### Isolation of CD19^+^ B cells, CD14^+^ monocytes and CD34^+^ progenitor cells

As a control group, B cells were isolated from blood samples of three patients with inflammatory diseases (cell group “BCI”) and from a buffy coat of a healthy donor (cell group “BC”). PBMCs were isolated over a Ficoll Hypaque gradient and purified with CD19 MACS MicroBeads. CD14^+^ blood monocytes from healthy individuals were isolated and differentiated as described previously [9]. Burst-forming unit-erythroid (BFU-E) and colony-forming units containing granulocytes and macrophages (CFU-GM) were generated from human CD34 progenitor cells as previously described [26].

### Mice

Male C57BL/6 mice and IL-7 receptor knockout mice (IL-7R^−/−^) were bred and maintained at the animal facility of the Medical University of Göttingen, Germany, according to the Deutsche Tierschutzgesetz (LAVES Niedersachsen 33.9.42502-05-A-08/09), after protocol review and approval by the committee of animal welfare of the University of Göttingen (“Tierschutzkommission”). After cervical dislocation, macrophages from the bone marrow were collected and purified by CD11b- MACS as described previously [10].

### Flow cytometry

Flow cytometric analyses were performed on a FACSCanto flow cytometer (BD Biosciences) as previously described [9,10] using the following antibodies: anti-human CD19-APC-H7, anti-human CD14-FITC, anti-human CD79b-APC and anti-human CD16-PE; anti-mouse CD11b-FITC and anti- mouse CD19-PE (all BD Biosciences).

### Immunoblotting

Immunoblot analyses were performed with standard techniques using the following antibodies: chicken anti-human IgA, chicken anti-human Igκ, mouse anti-human IgM, goat-human IgG, HRP. Proteins were extracted with TRI Reagent (Sigma) and dissolved in 1% SDS + 8M urea in 50mM Tris (pH 8.0) (vol/vol 1:1).

### Immunocytochemistry

Immunostaining was performed as described previously [10]. The following antibodies were used: Mouse anti-human antibodies to TCRβF1 (clone 8A3) (1:50, Thermo Scientific), rabbit anti-human IgM (1:50, acris). Goat anti-mouse IgG Alexa 488 labeled (Invitrogen), donkey anti-rabbit IgG, Cy3 (Jackson ImmunoResearch) were used as secondary antibodies. Mouse IgG1, rabbit IgG (BD Biosciences) isotype control antibodies were used as negative controls. For fluorescence imaging DRAQ5^TM^ (1:2000) (Alexis) was used for nuclear staining.

### RT-PCR, repertoire PCRs, CDR3 length spectratyping analysis and cloning

Total RNA from pooled purified CD14^+^ cells was reverse transcribed with oligo(dT) primers targeting mRNA. RT-PCR expression profiling was performed with primers for IgM, IgG heavy chains and κ and λ light chains. To establish Ig-repertoires, the collected RNA samples were reverse-transcribed into cDNA using the Reverse Transcription System (Promega). After PCR with the FastStart High Fidelity PCR System (Roche) and specific primers, PCR products were applied to an agarose gel and positive bands were excised. The PCR products were extracted using the QIAquick Gel Extraction Kit (Qiagen) and were subsequently cloned (TOPO TA Cloning Kit, Invitrogen) for individual sequence analyses. Size spectratyping of the antigen binding CDR3 regions were performed as previously reported [6,9]. Length variant analysis of the human and mouse Ig CDR3 regions were assessed using the D4-labeled primers D4-ccctgtccgctttcgctcca (human IgA), D4-tccttgaccaggcagcccag(human IgG1), D4-tgctgctgatgtcagagttg (human IgM) and D4 tcaaggatgctcttgggaga (murine IgM), respectively.

### Ig heavy chain locus recombination assay

DNA from 10^**6**^ monocytes was isolated using the Wizard Genomic DNA purification Kit (Promega). Screening for IgVH → IgJH rearrangements at the Ig heavy chain locus was performed by PCR utilizing a modified non-multiplex approach according to the protocols by van Dongen et al. [27] and confirmed by sequencing.

### Single cell analysis

Single cells were prepared using a protocol adapted from Kim et al. [28]. The cells were coated with CD14 Dynabeads and resuspended in PBS containing 2% BSA (~100 cells/ml). Under a microscope, single cells were aspirated with 0.5 μl medium and transferred into 3.5 μl resuspension buffer (5x RT buffer, OneStep RT PCR Kit, Qiagen). The reaction tubes were kept at -80°C until the next step. RT-PCR followed the protocol from Kim et al. [28] using the OneStep RT-PCR Kit (Qiagen) and specific reverse primers for IgM, IgG, Igκ and Igλ. Each reaction was then split into four aliquots and PCRs using specific forward and reverse primers for either IgM, IgG, Igκ or Igλ were performed following the protocols described above.

### Sanger sequencing and next-generation sequencing

Sanger sequencing was performed on an ABI Prism 310 one capillary sequencer. Large groups of samples were commercially sequenced by StarSEQ (Mainz, Germany). Obtained sequences were aligned and edited with MEGA 5.0 [29] and assigned the best matching germline variable region according to the integrative database VBASE2 [30]. A clonotype describes a unique sequence variant found in the repertoire. The results analyzed here only take functional rearrangements into account. For the next-generation sequencing approach the semi-quantitative technique of amplicon rescued multiplex (ARM)-PCR was used [31]. This technique relies on the use of universal primers at the exponential phase of PCR amplification to minimize target sequence-specific PCR amplification biases. Total TAM RNA was transcribed into cDNA using the OneStep RT PCR kit (Qiagen). The Ig transcriptomes were then amplified by ARM-PCR utilizing a set of nested Ig-specific Illumina primers provided by iRepertoire, Inc [32]. In the first amplification round (15 cycles), Ig heavy chain V segments were targeted specifically by nested forward and reverse primers. A pair of common sequence tags was linked to all internal primers, which were targeted in the second round of amplification (40 cycles) by universal primers. PCR samples were subsequently purified by gel electrophoresis and sent to iRepertoire for NGS. Large-scale sequencing of was performed on a HiSeq2000 DNA sequencer (Illumina) using the standard Illumina sequencing protocols. The NGS data has been deposited in the NCBI Sequence Read Archive (SRA) (Accession #SAMN09662874, SAMN09662875, SAMN09662876).

### Alignment of immunoglobulin repertoire sequences and data filtering

Complementary determining regions (CDR3s) were defined as the segment that encompasses all amino acids flanked by the conserved amino acid sequences Y[YFLI]C at the 3′end of the V gene segment and [FW]GXGT (X stands for 1 of 20 amino acids) within the J segments. Raw data was analyzed by iRepertoire using the previously described IR map program [32]. The best matches of germline V and J gene were searched by determining alignments between Illumina platform product and germline sequences in the IMGT/GENE-DB database.

Data analysis was conducted using the newly developed five-step SMART filtering strategy This advanced computational strategy relies on a cascade of filtering algorithms that eliminate artifactual sequences at five distinct quality control checkpoints: (i) sequencing error filter, (ii) mosaic sequencing filter, (iii) PCR amplification performance filter, (iv) reference sequence filter and (v) frequency threshold filter, respectively. All mapped CDR3 reads were subjected to the SMART strategy and the frequency threshold filter was set to >1. Under these conditions all single copy Ig heavy chain CDR3 sequence variants were disregarded.

### Statistical analysis

The Shannon-Wiener index [33,34] was used to compare the significance of repertoire diversity between cell groups. p<0.001 was considered statistically significant.

## Results

### Expression of immunoglobulins in monocytes/ macrophages

**Fig 1:**
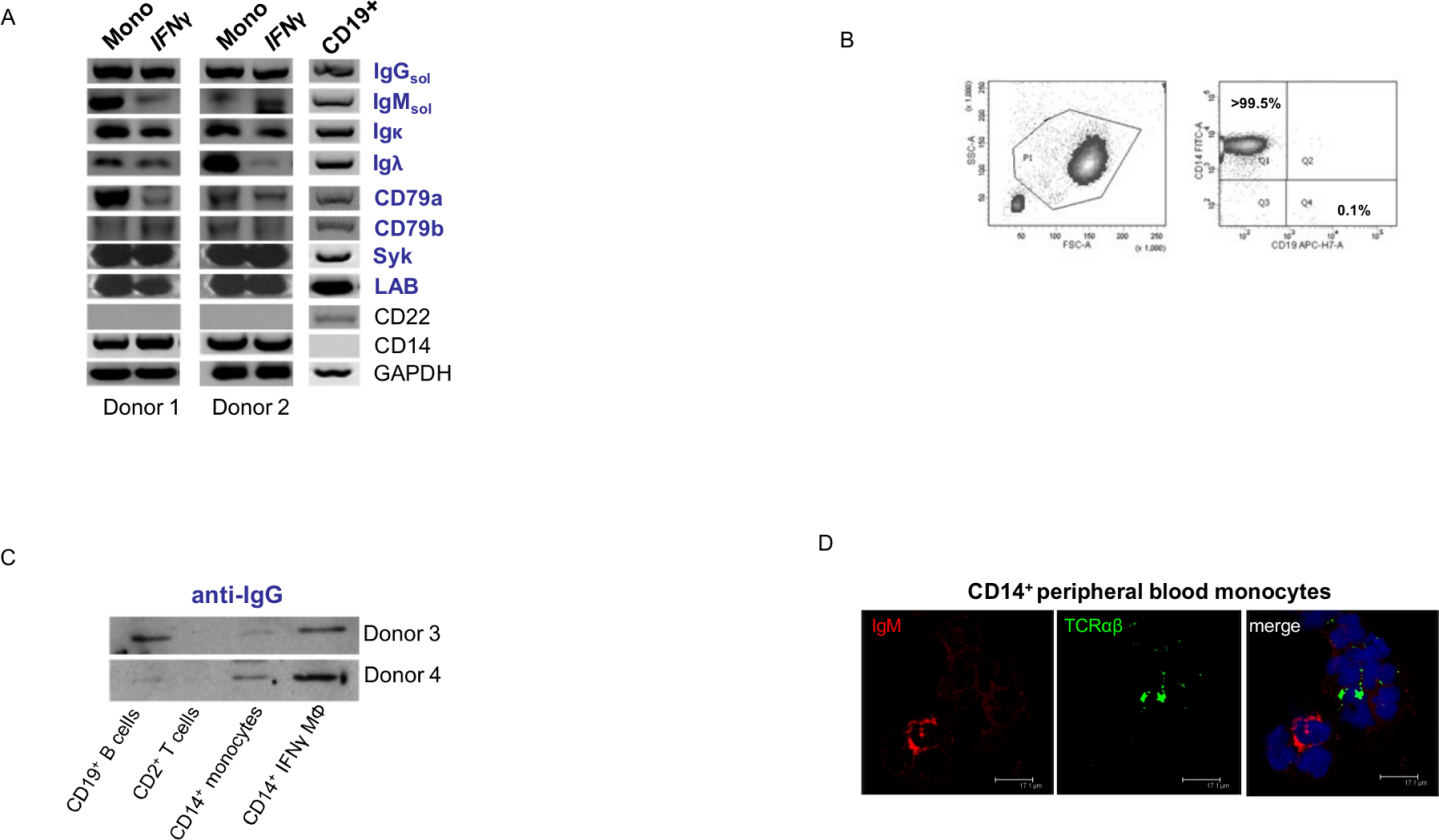
Human monocytes and macrophages isolated from healthy individuals express immunoglobulin heavy and light chains. **(A)** Circulating human monocytes (Mono) and IFNγ activated macrophages (IFNγ),respectively, constitutively express the genes for the Ig heavy and light constant chains (IgM, IgG, Igκ, Igλ) and critical components of the Ig signalling complex (CD79a, CD79b, Syk, LAB). RT-PCR profiling is shown for CD14^+^ monocytes and IFNγ *in vitro* differentiated macrophages from two representative healthy individuals and CD19^+^ B cells from an additional healthy donor. Expression of B cell (CD22) and monocyte/macrophage marker genes (CD14) are demonstrated as reference. CD79a, CD79b B-cell antigen receptor complex-associated protein alpha and beta chain; Syk spleen tyrosine kinase; LAB linker for activation of B cells. **(B)** Flow cytometry of freshly isolated MACS purified CD14^+^ monocytes representatively shown for one individual demonstrates purity of routinely >99.5%. The lineage surface markers CD14 and CD19 are shown. **(C)** Detection of immunoglobulin IgG in monocytes and macrophages by immunoblot. Whole cell lysates of CD14^+^ monocytes, IFNγ-activated CD14^+^ macrophages, CD19^+^ B cells and CD2^+^ T cells from donors 3 and 4 were separated under reducing conditions. An antibody against IgG was used for detection. **(D)** Immunocytochemical double-staining reveals the presence of TCRβ (green) and IgM (red) expressed by two separate subpopulations of CD14^+^ monocytes. Shown is a IgM^+^ monocyte (left) next to a TCRβ^+^ monocyte (right) surrounded by double- negative monocytes. The results are representative of three individuals. Nuclei (blue), DRAQ5. Isotype controls for the anti-ΤCRαβ and anti-IgM antibodies are shown in the supplements (S3 Fig).

In preliminary assessments we tested whether the monocytic lineage expresses immunoglobulins (Ig). For this human peripheral blood CD14^+^ monocytes were obtained from two healthy donors and monocyte differentiation into Th1-polarized macrophages was induced in the presence of IFNγ. In fact, RT-PCR profiling of these cells demonstrated constitutive expression of Ig heavy and light chains, but also critical components of the Ig signaling complex in both CD14^+^ monocytes and IFNγ macrophages (Fig 1A). Similar results were obtained with the Th2-stimulus IL-4 (S1 Fig). The high purity (>99.5%) of isolated CD14^+^ monocytes (Fig 1B) and consistent absence of expression of the B cell marker CD22 indicated that these results were not attributable to B cell contamination.

Consistent with the existence of a *bona fide* Ig^+^ human macrophage subpopulation, immunoblot revealed the presence of both Ig heavy (IgM, IgG and IgA) and Ig light chains (Igκ) in the IFNγ *in vitro* differentiated macrophages from the two healthy donors (Fig 1C; S2 Fig).

Next, we determined expression of Ig in individual circulating monocytes. For this, freshly isolated CD14^+^ monocytes were stained for IgM. We detected IgM expression in a small (~5%) monocyte subpopulation (Fig 1D; S3 Fig). Of note, this subfraction was clearly distinct from the previously reported TCRαβ bearing monocyte/macrophage subpopulation [9] as evidenced by co-staining with antibodies to TCRαβ. Further analysis of the respective subpopulation points to the highest immunoglobulin expression in classical monocytes (CD14^+^/CD16^-^) (S4 Fig).

Together, these results reveal that subpopulations of peripheral blood monocytes and *in vitro* activated monocyte-derived macrophages express immunoglobulins both on the gene expression and protein level.

### Human and murine macrophages express variable immunoglobulin heavy chain repertoires

**Fig 2:**
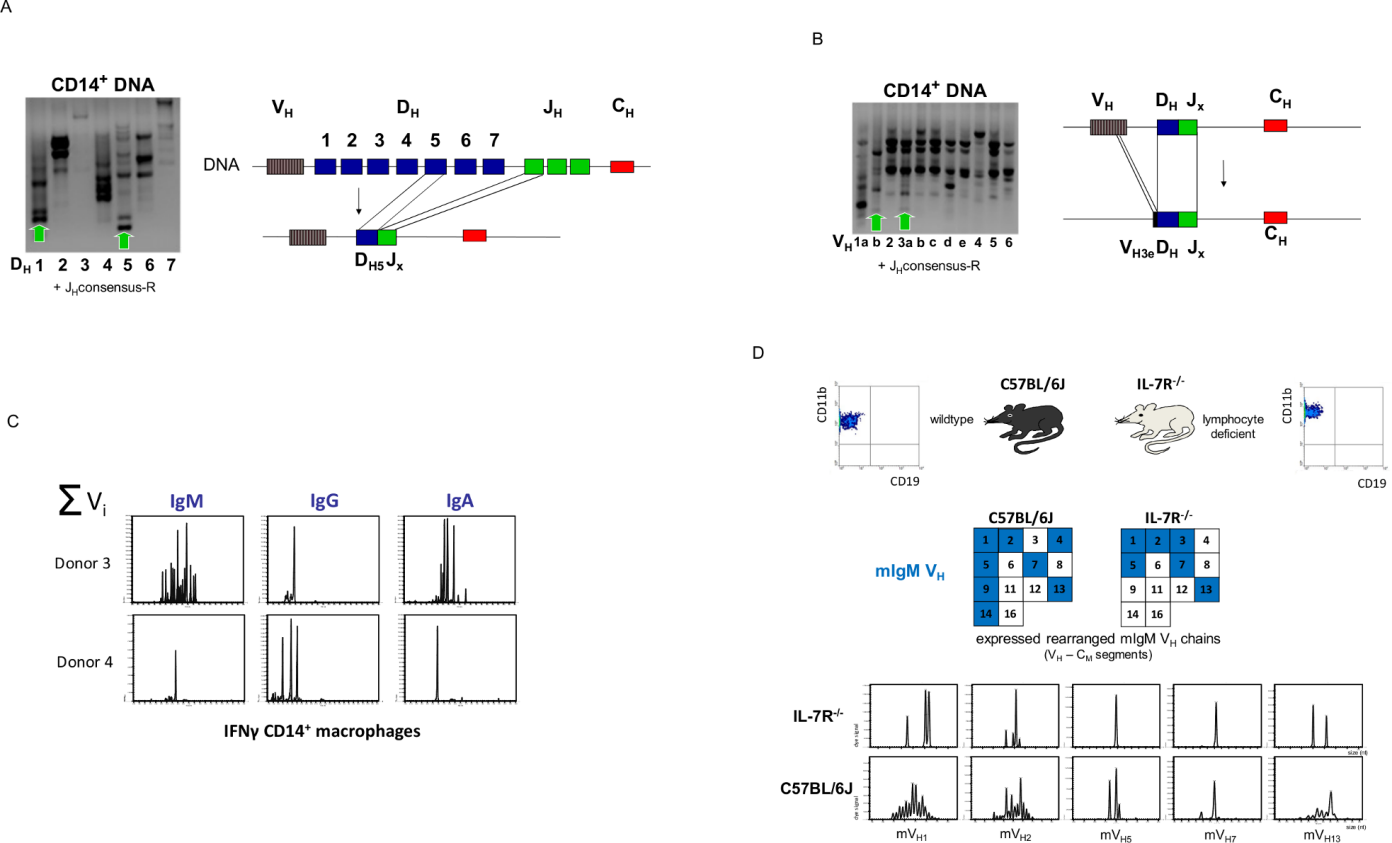
Monocytes and macrophages express rearranged immunoglobulins. Detection of D → J **(A)** and V → DJ **(B)** rearrangements in the Ig heavy chain gene locus of human CD14^+^ monocytes. Arrows denote the presence of D_H_1, D_H_5→ J and V_H_1b, 3a→ J rearrangements which were confirmed by sequencing. Genomic organization of the identified rearrangements is schematically drawn. **(C)** Length variant analysis of the antigen-binding complementarity determining region 3 (CDR3) demonstrates the constitutive expression of individual-specific IgM, IgG and IgA variable heavy chain repertoires, respectively, by IFNγ macrophages from two healthy donors (donor 3 and 4). V_H_−C_M/G/A_ specific cDNA segments were amplified by RT-PCR and separated by capillary electrophoresis (“CDR3 spectratyping”). A scaled synopsis of all expressed V chains is shown (∑). The detailed CDR3 length spectratypes for each expressed V chain are shown in the supplement (S5 Fig). **(D)** Profiling of IgM V heavy chain expression in bone marrow CD11b^+^ macrophages from wildtype mice (wt) and mice lacking lymphocytes (IL-7 receptor knockout mice, IL-7R^-/-^). FACS analysis shows the purity of the isolated CD11b^+^ bone marrow macrophage fractions. All macrophages were purified by CD11b-MACS. IgM V_H_ chain gene expression was assessed by RT-PCR amplification of rearranged V_Hi_−C_M_ cDNA segments. Expressed V_H_ chains are highlighted by filled boxes, empty boxes represent nonexpressed V_H_ chains. Numbers denote individual V_H_ chains. The detailed CDR3 length spectratypes are shown for the five V_H_ chains that are expressed by both wildtype and IL-7R^-/-^ mice (bottom). Note the reduced repertoire diversities in lymphocyte-deficient IL-7R^-/-^ mice relative to wildtypes. The detailed CDR3 length spectratypes for all expressed V chains are shown in the supplement (S6 Fig).

Based on the results demonstrating rearrangement of the TCRβ locus in human monocytes/ macrophages and evidence of RAG1/2 and TdT in these cells [10], rearrangement of the Ig heavy chain locus in monocytes was investigated. The first recombination event at the Ig heavy chain locus juxtaposes one D and one J gene segment, with deletion of the intervening DNA followed by the second step of Ig heavy chain rearrangement, i.e. V→ DJ recombination giving rise to a fully rearranged VDJ variable region gene. A modified PCR assay based on the method by van Dongen et al. [27] was applied. In fact, the PCR assay revealed recombination for D_H_1 and D_H_5, respectively, using the J consensus primer reported by van Dongen et al. [27] and V_H_1b and V_H_3a → DJ rearrangements in normal human CD14^+^ monocytes (Fig 2A and 2B). Together, these experiments provide evidence for both D→ J and V→ DJ rearrangements of the Ig heavy chain locus in normal peripheral blood monocytes.

The analysis of all expressed CDR3 length variants for IgM, IgG and IgA, respectively, in human IFNγ macrophages from two healthy individuals demonstrated constitutive expression of the immunoglobulin isotypes (Fig 2C, S5 Fig). We found that the expressed immunoglobulin repertoires are complex and individual-specific suggesting that the immunoglobulin isotypes are rearranged and expressed in macrophages.

To apply our findings in humans to a controlled experimental system, we isolated CD11b^+^ macrophages from the bone marrow of lymphocyte-deficient IL-7R^-/-^, and C57Bl/6 mice and analyzed the IgM heavy chain repertoires. The former were used as biological model organism from which B cells are substantially reduced to approximately <1% of wildtype C57Bl/6 mice [35,36]. In accordance with the human findings, we saw variable IgM expression in bone marrow macrophages from both IL-7R^-/-^ mice lacking lymphocytes and normal C57Bl/6 mice (Fig 2D, S6 Fig).

Taken together, the combined results from Ig VDJ rearrangement analysis and CDR3 mRNA expression profiling indicate that a subpopulation of monocytes/macrophages expresses recombinatorial Ig heavy chains in both humans and mice.

### Myeloid progenitors express recombinatorial immunoglobulins

**Fig 3:**
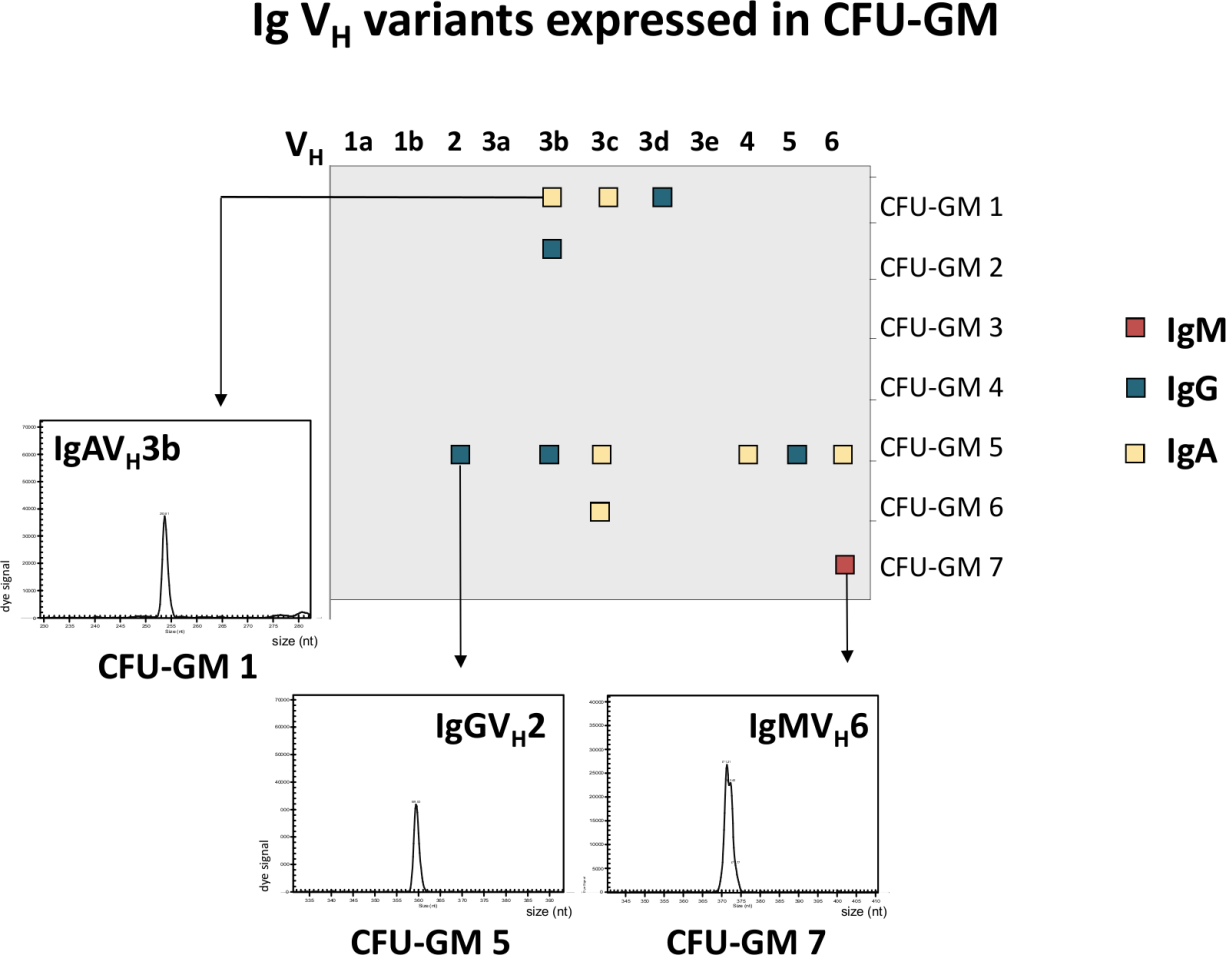
Myeloid progenitors express variable immunoglobulins. Expression of rearranged IgM, IgG and IgA V_H_ CDR3 clonotypes in granulocyte/ macrophage progenitor colonies (CFU-GM) obtained from CD34^+^ progenitors of a healthy individual. Filled boxes indicate positive expression of one of the 11 known human Ig V_H_ chains (x-axis) in a single colony. Colonies are identified by numbering on the y-axis. The repertoires for each of the expressed V_H_ chains were determined by CDR3 spectratyping. Three detailed V_H_ repertoires are representatively shown. The repertoires of additional CFU-GM colonies are summarized in the supplement (S7 Fig).

Evidence for Ig locus rearrangement in mature CD14^+^ monocytes and reports of immunoglobulin expression in CD34^+^ progenitor cells [37] strongly suggested that Ig recombination might already occur at an early stage of myeloid development. To test the variability of myeloid Ig at this early developmental stage, CD34^+^ hematopoietic progenitor cells were stimulated by GM-CSF and subsequently formed colony forming units (GM-CFU). IgG, IgM and IgA V mRNA expression profiling was performed on 7 randomly selected colonies. CDR3 length spectratyping revealed expression of single or few rearranged V heavy chain clonotypes (1 IgM, 5 IgG and 6 IgA, respectively) (Fig 3, S7 Fig). The majority of the CFU-GM displayed a monoclonal expression pattern consistent with the clonogenic nature of the myeloblasts and monoblasts in this assay. This confirms that Ig locus rearrangement occurs already during the early phase of *in vitro* myeloid lineage differentiation.

### Tumor-associated macrophages express restricted Ig repertoires

**Fig 4:**
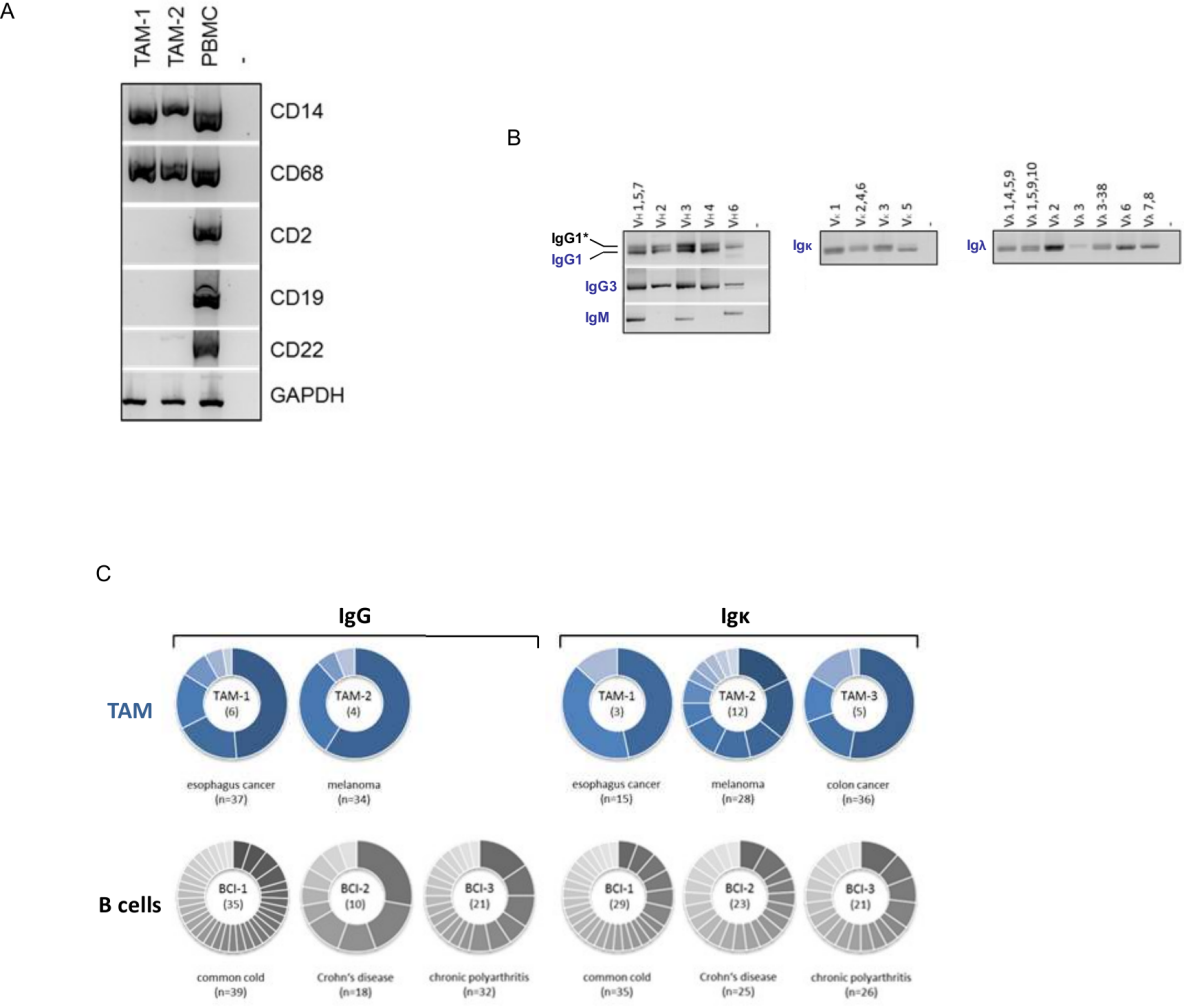
Tumor-associated macrophages express restricted Ig repertoires. **(A)** RT-PCR expression profiling of TAM cell preparations for B cell (CD19, CD22), T cell (CD2) and monocyte/ macrophage marker genes (CD14, CD68), respectively, demonstrates absence of detectable quantities of B or T cells. The results shown for tumor patients TAM-1 and TAM-2 are representative of all TAM cell preparations. PBMC, peripheral blood mononuclear cells (positive control); -, no template added. **(B)** CD14+ macrophages isolated from a human melanoma patient (TAM-2) express combinatorial heavy (IgG1, IgG3, IgM) and light chain genes (Igκ,Igλ). Diagnostic amplification products span V_H_−C, and V_κ_-C and V_λ_-C segments, respectively, and were generated using consensus primers for the indicated variable chains (V_H_i, V_κ_j, V_λ_i). -, no template added. Note expression of an additional IgG1 transcript (IgG1*, +97 bp) which is generated by intron retention within the C_H_ gene and gives rise to an alternative C-terminus (unpublished data). **(C)** IgG and Igκ repertoire diversities in tumor macrophages from patients TAM-1 to TAM-3. Sector areas correspond to the relative frequency of individual clonotypes within a given Ig CDR3 repertoire and are sorted by size. Each sector within a circle chart represents a unique CDR3 variant and the number of identified variants is shown in the center. Clonotype frequencies in B cells from patients with acute and chronic inflammatory diseases are shown for reference (BCl-1, BCl-2, BCl-3). Note that TAM express less diverse IgG and Igκ repertoires than B cells from patients with inflammatory diseases. The total number of Sanger sequenced clonotypes are shown at the bottom of each pie chart.

Recent evidence revealed the presence of TCRαβ bearing macrophage subpopulations in the tumor microenvironment [15]. We therefore investigated next whether macrophage-derived Ig are also implicated in tumor-associated inflammation. To address this, we first tested whether TAM from freshly excised human tumor specimens express combinatorial Ig heavy and light chains. For this, purified TAM from a human esophagus carcinoma (TAM-1) and a melanoma patient (TAM-2) were freshly obtained. Using RT-PCR analysis for leukocyte lineage markers, B lymphocytic cells were undetectable in the tumor macrophage preparations (Fig 4A). Recombination analysis of expressed V_H_−C, V_κ_-C and V_λ_-C segments, respectively, revealed that a variety of rearranged Ig heavy chains (IgG1, IgG3, IgM) and also Igκ and Igλ light chains were detectable in the melanoma-derived TAM (Fig 4B). This demonstrates that macrophages in the tumor microenvironment exhibit Ig combinatorial diversity.

### TAM Ig heavy and light chain repertoires are less diverse than those of B cells

We then performed a detailed profiling of the CDR3 heavy and light chain Ig repertoires expressed by TAM obtained from the patients with esophagus cancer, melanoma and colon cancer, respectively, (TAM-1, TAM-2, TAM-3) (Table 1). To this end, the IgM, IgG, Igκ and Igλ repertoire diversities were assessed by cloning and Sanger sequencing of the expressed Ig CDR3 variants. Peripheral blood B cells from three acute and chronic inflammatory diseases including common cold (BCl-1), Crohn’s disease (BCl-2) and chronic polyarthritis (BCl-3) were co-analyzed as reference. Using this approach, we identified 6 distinct IgG heavy chain CDR3 variants and 3 Igκ variants in the esophagus cancer-derived TAM-1 (Fig 4C, Table 2, S1 Table). In the melanoma-derived TAM-2 a total of 4 IgG CDR3 variants and 12 Igκ CDR3 variants were detected whereas only Igκ variants (n = 5) were identified in the colon cancer tumor macrophages (TAM-3). In general, the B cell populations from the patients with acute and chronic inflammation exhibited significantly higher repertoire diversity ranging from 10–35 (IgG) and 21–29 (Igκ) CDR3 variants, respectively (S8 Fig).

**Table 1:** Characteristics of the tumor-associated macrophages (TAM), B cells from patients with inflammatory diseases (BCI) and normal B cells (BC) used in this study.

Patient	Age	Sex	Medical condition	Isolated cells	Source	Cell type
**TAM-1**	63	F	Esophagus carcinoma	Macrophages	Solid tumor	**TAM**
**TAM-2**	73	M	Melanoma	Macrophages	Solid tumor (metastasis)	
**TAM-3**	63	M	Adeno-carcinoma	Macrophages	Solid tumor	
**TAM-4**	unk.	unk.	Colorectal carcinoma	Macrophages	Solid tumor	
**TAM-5**	unk.	unk.	Colorectal carcinoma	Macrophages	Solid tumor	
**TAM-6**	62	F	liver carcinoma	Macrophages	Solid tumor	
**TAM-7**	77	M	Colorectal carcinoma	Macrophages	Solid tumor	
**TAM-8**	25	M	lymphoma	Macrophages	Lymphoma filtrate	
**TAM-9**	76	F	Colorectal carcinoma	Macrophages	Solid tumor	
**TAM-10**	47	W	glioblastoma	Macrophages	Solid tumor	
**BCI-1**	23	F	Common cold	B cells	Peripheral blood	**BCI**
**BCI-2**	43	M	Crohn’s disease	B cells	Peripheral blood	
**BCI-3**	62	M	Chronic polyarthritis	B cells	Peripheral blood	
**BC**	unk.	unk.	-	B cells	Peripheral blood	**BC**
**BC-2**	25	M	-	B cells	Peripheral blood	

F = female, M = male; TAM = Tumor- associated macrophages; BCI = B cells of patients with inflammatory disease; BC = B cells of healthy controls; unk. = unknown

**Table 2:** Total number of clones analyzed for each patient and repertoire.

Cell type	Patient	IgM	IgG	Igκ	Igλ
**TAM**	TAM-1	34 (0)	38 (1)	15 (0)	
	TAM-2	40 (1)	34 (0)	34 (6)	36 (0)
	TAM-3	53 (2)		40 (4)	
**BCI**	BCI-1		39 (0)	37 (2)	
	BCI-2		18 (0)	26 (1)	
	BCI-3		34 (2)	31 (5)	
**BC**	BC		34 (1)	35 (0)	

Sequences which contained stop codons or frameshifts are in parentheses and were not taken into account for further analyses.

The most frequent Ig heavy chain CDR3 variant was ARVPINYDILTGTDY which was expressed both by esophagus cancer TAM and melanoma-derived TAM. Interestingly, this variant was the only one that was shared by distinct TAM populations. In contrast, CDR3 variant sharing was more frequently observed for the Igκ light chain repertoires. The most frequent Igκ variant, QQYNTYPLT, for example, was expressed both by TAM-2 and B cells from the patient with common cold (BCI-1) (S2 Table).

The marked restriction of the TAM Ig repertoires was striking. V_H_4-61/D2-2/J_H_3 and V_H_4-31/D3-3/J_H_4 were the most common VDJ heavy chain recombinatorial variants which were present 20 findings in TAM-1 (49%) and TAM-2 (59%), respectively. None of the B cell heavy chain repertoires showed a similar restriction with the most frequent rearrangements ranging between 6% and 12% (S3 Table, S4 Table).

### Profiling of TAM Ig heavy chain repertoire transcriptomes by ultra-deep sequencing

**Fig 5:**
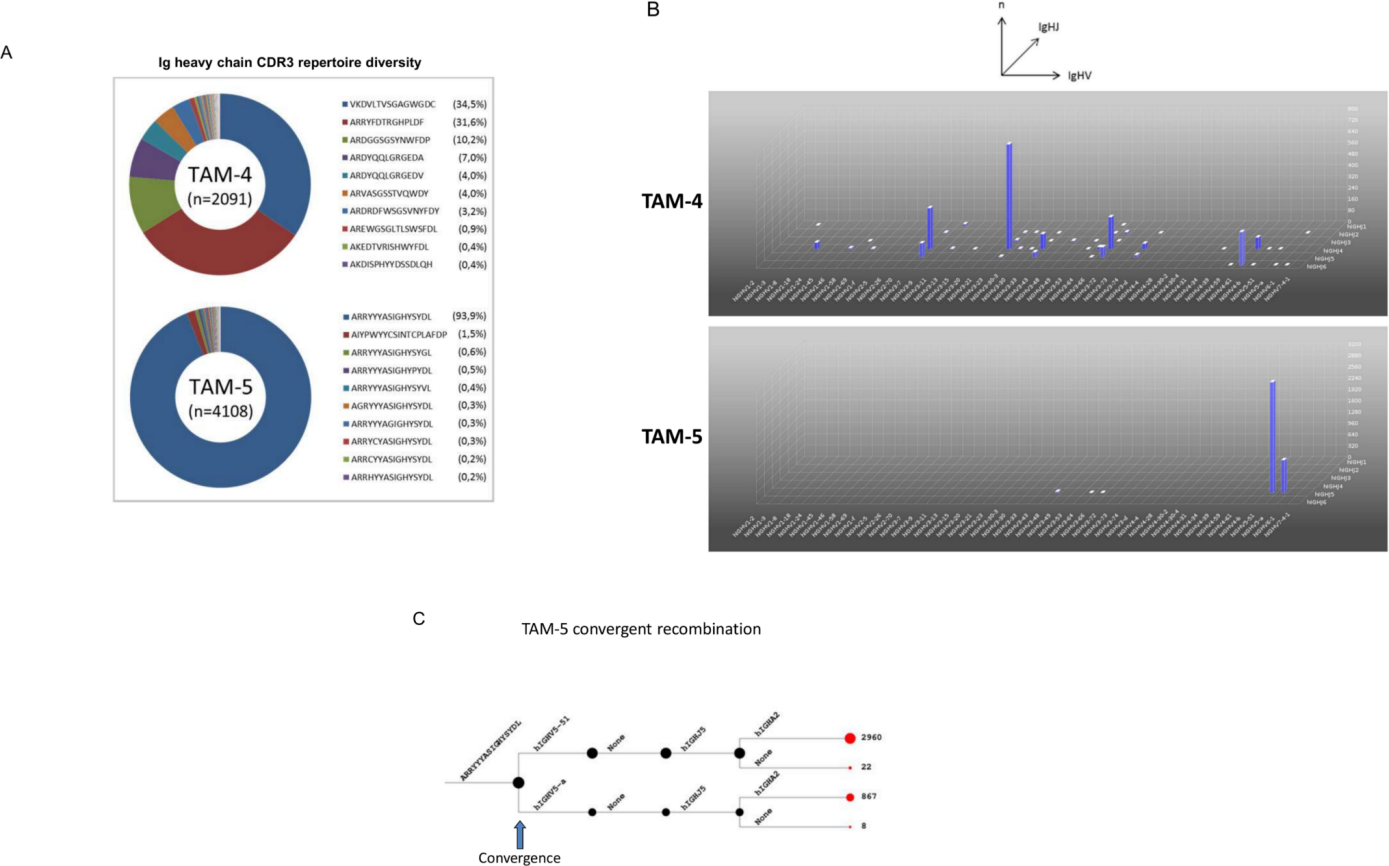
TAM Ig heavy chain repertoire transcriptome profiles assessed by ultra-deep sequencing. (**A**) Shown are the Ig V_H_ CDR3 repertoires of TAM isolated from two patients with colorectal cancer (TAM-4, TAM-5) as assessed by ultra-deep sequencing. The circle graphs visualize the relative abundance of the Ig heavy chain CDR3 transcript variants that are expressed by each TAM population. Each sector in a circle represents a rearranged Ig heavy chain transcript that encodes a unique CDR3 sequence. It is defined by a unique color and its area is proportional to the relative transcript frequency. The total numbers of identified Ig V_H_ CDR3 variants are indicated. A CDR3 sequence is considered unique if it represents a nonredundant fragment of amino acids, which is in a stop-codon-free reading frame and contains both translated conserved V_H_ and J_H_ motifs. The 10 most frequently expressed Ig heavy chain CDR3 variants in each TAM population are and their respective percentage (in parentheses) are shown. **(B)** IgHV and IgHJ gene usage by TAM-4. The 3D expression frequency maps display the relative transcript abundance for each expressed IgHV_x_/IgHJ_x_ combination. Individual human Ig heavy chain V-genes and J-genes (hIGHV_x_, hIGHJ_y_) are annotated according to the nomenclature used by the IMGT/GENE database (http://www.imgt.org/IMGTindex/IMGTgene-db.html). X-axis: IgHV gene. Y- axis: IgHJ gene. Z-axis: relative expression frequency. (**C**) Evidence for convergent recombination of a TAM Ig heavy chain CDR3 region (patient TAM5). Shown is the differential use of the human IGHV5-51 and IGHV5-a genes which converge to produce the same Ig heavy chain CDR3 variant (ARRYYYASIGHYSYDL) (arrow). This CDR3 variant was identified by deep sequencing. Total numbers of sequence reads representing this CDR3 variant are indicated (right).

To substantiate the above results obtained by conventional cloning and sequencing on a large scale level, we analyzed two tumor macrophage populations from patients with colon cancer (TAM-4, TAM-5) utilizing the highly sensitive technique of ultra-deep sequencing. For this, TAM-4 and TAM-5 were subjected to ARM-PCR based Ig heavy chain transcriptome sequencing [31,32]. This resulted in 6284 effective sequence reads for both samples which could be assigned to 150 unique IGH CDR3 segments (S5 Table, S9 Fig). A unique IGH CDR3 sequence was defined as a nonredundant fragment of amino acids which is in a stop-codon-free reading frame and contains both translated conserved V_H_ and J_H_ motifs. To correct for sequencing artifacts all sequence reads were subjected to high-stringency filtering algorithm with the transcript frequency threshold set to >1. This conservative approach, in which all single copy CDR3 variants were *a priori* disregarded, resulted in the identification of a total of 65 unique Ig heavy chain CDR3 variants from the TAM subpopulations of the two donors. As a reference, we performed transcriptome sequencing under identical conditions on peripheral blood CD19^+^ B cells from a healthy donor which resulted in the identification of 25.827 unique Ig heavy chain CDR3 variants (S10 Fig). In both colon cancer TAM populations we noted that the distribution of the CDR3 clonotypes was markedly skewed with only a few CDR3 clonotypes making up the majority of all CDR3 transcripts. The most frequently expressed Ig heavy chain CDR3 transcript variant in the TAM-4 population was VKDVLTVSGAGWGDC (34%), followed by ARRYFDTRGHPLDF (32%) and ARDGGSGSYNWFDP (10%), respectively (Fig 5A). The dominance of a single heavy chain variant was even more striking in the TAM-5 population in which the clonotype ARRYYASIGHYSYDL represented 94% of all identified CDR3 Ig heavy chain transcripts (S5 Table).

To identify shared Ig heavy chain CDR3 sequences, we performed pairwise bioinformatic comparisons between the TAM-4 and TAM-5 Ig transcriptomes. Interestingly, we found that no CDR3 sequences were shared between the two TAM Ig heavy chain repertoires. Moreover, none of the TAM Ig heavy chain CDR3 variants that were expressed in the two colon cancer tissues were identified in the other sequenced repertoires (TAM-1, TAM-2, TAM-3). Together, this suggests that the Ig heavy chain variants in TAM populations are expressed in an individual-specific fashion.

Detailed comparison of the TAM-4 and TAM-5 V-J usage and the frequency of individual CDR3 transcript variants revealed highly restricted and unique repertoire diversity patterns with a striking bias towards the use of single V and J genes. We found that the TAM-4 population predominantly used IgVH3-23 and IgJH4 (Fig 5B). Interestingly, these are also the most utilized Ig V-J heavy chain genes in B cells [38]. Conversely, the macrophages from the second colon cancer (TAM-5) relied mostly on the usage of IgVH5-51 and IgJH5 (S11 Fig). Convergent recombination is a mechanism by which divergent Ig variable heavy chain sequences are assembled in B cells to produce identical CDR3 at the amino acid level [38,39]. To test whether this mechanism is also operative in TAM, we performed a detailed VDJ recombination analysis in TAM-5. Indeed, we found evidence for the differential use of the IGHV5-51 and IGHV5-a genes which converged to form an identical Ig heavy chain CDR3 variant (ARRYYYASIGHYSYDL) (Fig 5C).

### Ig clonotype analysis in single macrophages

Given the above combined evidence for Ig production by macrophages, we finally sought to assess in detail the actual pairing of the Ig heavy and light chain in a single macrophage.

For this, we performed the single cell Ig clonotype analysis on five different tumor tissues including a liver carcinoma, a glioblastoma, a lymphoma and two colon cancer (TAM-6 –TAM-10). From these a total of 102 single tumor macrophages were subjected to expression profiling of the IgG, IgM, Igκ and Igλ chains, respectively, followed by cloning and sequencing. Out of this pool of single TAM we were able to identify eight Ig complete heavy chain sequences and two light chain sequences (S6 Table).

**Fig 6:**
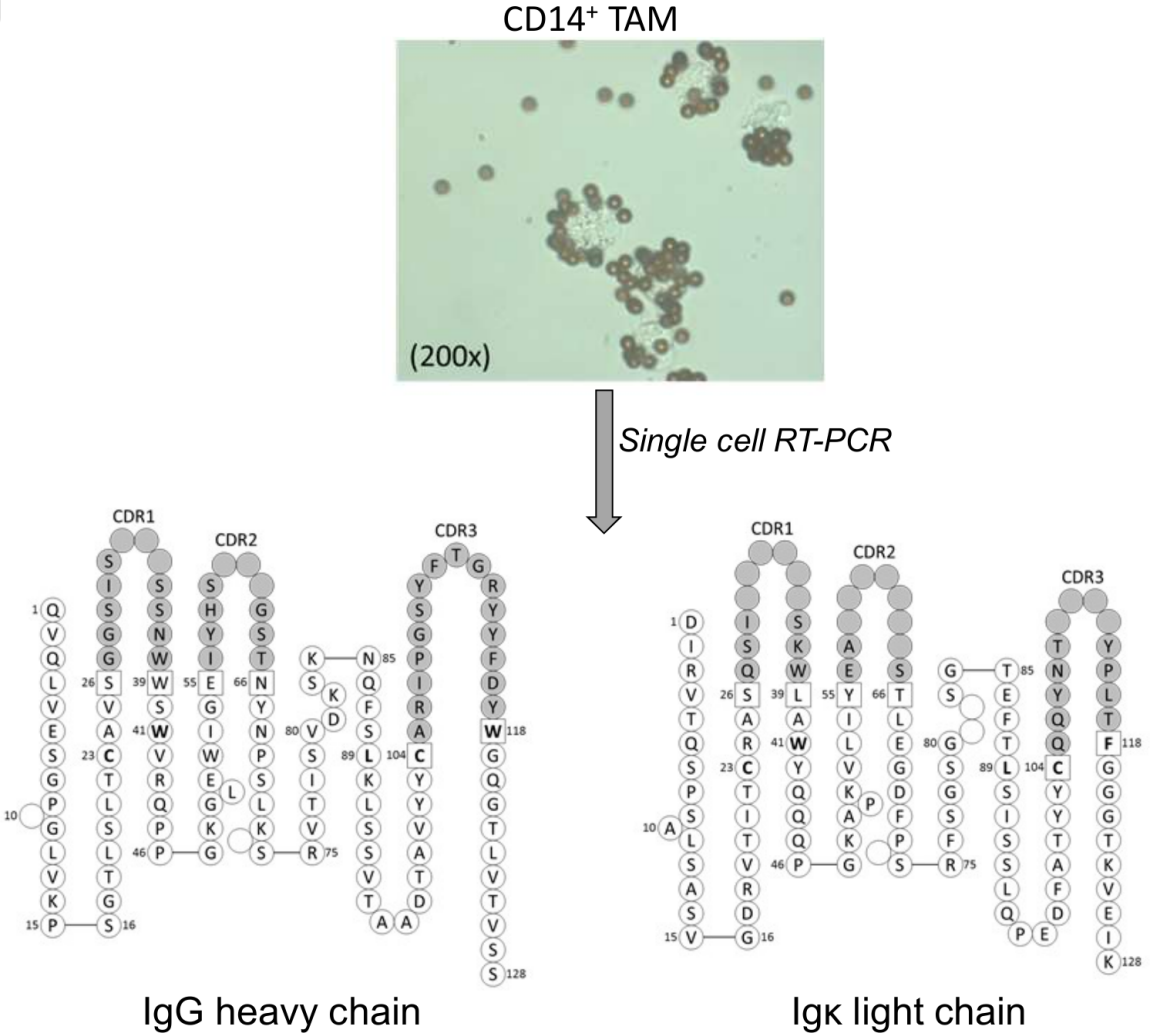
Single cell Ig clonotype analysis in a tumor-associated macrophage. **(Top)** CD14^+^-MACS macrophages purified from tumor tissue of a patient with esophagus cancer (TAM-1). The light microscopic image shows anti-CD14^+^ coated magnetic beads attached to four individual macrophages. CD14^+^ bead positive cells were subsequently subjected to single cell Ig clonotype analysis. **(Bottom)** Complete IgGκ clonotype expressed by a single tumor-associated macrophage isolated from the above patient. The detailed peptide sequences of the IgGκ heavy and corresponding light chain are shown. The major antigen-binding CDR3 regions are gray shaded. Single cell Ig clonotype analysis was assessed by single cell RT-PCR, cloning and subsequent Sanger sequencing.

In addition, we analyzed 424 single tumor macrophages that were isolated from esophagus cancer tissue (patient TAM-1). This resulted in the identification of a complete Ig clonotype which encoded a functional IgG heavy and a corresponding Igκ light chain (Fig 6). This Ig clonotype was detected in three distinct single tumor macrophages. The IgG heavy chain of this TAM clonotype was generated by a V_H_4-4/D1-26/J_H_4 VDJ rearrangement and the associated κ light chain displayed a V_κ_1-5/J_κ_4 rearrangement. We also identified three additional complete IgG heavy chain sequences in our TAM-1 single cell clonotype analysis. Two of these were identical to those for which the heavy-light chain pairing could be established and one utilized an ORF V gene. Furthermore, we clonotyped a functional IgM chain, however, we were unable to detect the corresponding light chain (data not shown). Together, these single cell expression profiling results demonstrate that tumor macrophages are capable of producing functional Ig heavy and light chain clonotypes.

## Discussion

This study demonstrates, for the first time, constitutive expression of combinatorial Ig heavy and light chains in (i) human peripheral blood monocytes, (ii) *ex vivo* differentiated macrophages and (iii) macrophages from the tumor micromilieu. Furthermore, we describe the first complete molecular structure of a macrophage-derived immunoglobulin at the single cell i.e. monoclonal level. Our results rely on combined evidence from RT-PCR, DNA rearrangement analysis, immunoblot, immunocytochemistry, Ig CDR3 length spectratyping, deep-sequencing based Ig transcriptome profiling and single cell Ig clonotype analysis, respectively. Moreover, we show expression of combinatorial Ig in murine macrophages.

Traditionally, production of variable immune receptors has been regarded as a unique feature of T and B lymphoid cells. However, recent evidence has accumulated during the past decade that indicates the expression of combinatorial immune receptors outside the lymphoid lineage. For example, combinatorial T cell receptor expression has been shown for human neutrophil, eosinophil, monocyte and macrophage subpopulations in the context of inflammatory diseases such as atherosclerosis, tuberculosis, periodontitis and cancer [6–15]. Moreover, previous studies have reported Ig heavy and/or light chain expression outside the realm of B lymphoid cells in malignant cells including human epithelial cancer cells [40], nasopharyngal carcinoma cells [41], leukemia cells [42] and diverse tumor cell lines [43–45]. In this context, Ig synthesis has largely been considered an aberrant feature of transformed somatic cells. However, recent studies in neuronal cells [46], epithelial cells [47], epidermal cells [48] and endothelial cells [49] suggest that non-transformed cell types are also capable of “ectopic” immunoglobulin production.

The widely accepted strict restriction of immunoglobulin production to lymphocytes and that of phagocytosis capacity to myeloid cells is further challenged by the discovery of B1 cells [50], NKB cells [51] and B1 phagocytes [52]. The latter are immunoglobulin-expressing phagocytic cells and are believed to originate from B1 cells. Recent evidence demonstrating the existence of a CD11b^+^ immunoglobulin-expressing B1 cell subpopulation further challenges the existing dogma [53]. However, the biological function of these cell populations is currently unclear.

Intriguingly, the recent observation by Huang et al. [16] and Wang et al. [17] that CD33^+^ monocytes and neutrophils from healthy individuals and patients with solid tumors express IgM heavy chains and IgK light chains provided an initial clue for Ig production by myeloid cells. However, these studies relied solely on gene expression profiling. Indeed, these studies confirm our work on the observation that monocytes express IgM and IgK. In addition, we show that both monocytes and macrophages from healthy individuals are capable of expressing IgG or IgA heavy chains and also Igλ light chains. These results indicate that production of Ig heavy and light chains is a genuine and physiological feature in a subset of monocytes and macrophages. Consistent with this, evidence for Ig synthesis was also found in murine macrophages, even in the absence of B and T cells in macrophages from genetically modified mice. Importantly, using single cell analysis, we were able to identify the complete heavy and light chain clonotype of a specific immunoglobulin expressed by a macrophage isolated from the tumor microenvironment. Together, these findings provide formal evidence that myeloid cells are indeed capable of producing functional immunoglobulins. Moreover, it will be challenging to identify the specific epitope(s) that are recognized by this macrophage-derived antibody.

During B cell development, the Ig gene segments are rearranged to yield an enormous repertoire of antigen-binding receptors. Here, DNA rearrangement assays, CDR3 spectratyping, conventional Sanger sequencing and deep sequencing analyses of human and murine immunoglobulins from monocytes/ macrophages confirm that the immunoglobulin heavy and light chains underwent successful genomic V(D)J rearrangement. Interestingly, these repertoire diversity analyses of *ex vivo* differentiated macrophages and tumor macrophages revealed that macrophages express markedly restricted Ig repertoires relative to normal B cells or B cells from patients with inflammatory diseases. Furthermore, the macrophage Ig repertoires show a biased V- and J chain usage and are individual-specific consistent with the expression of a few dominant clonotypes. The phenomenon of restricted Ig repertoires has also been described in other non-B cell populations for example in neuronal cells [46] or endothelial cells [49]. Importantly, Huang et al [16] and Wang et al [17] also reported on marked restriction in variability of IgM and IgK expression in myeloid cells. Likewise, TCR repertoires expressed by macrophages and neutrophils display a similar repertoire restriction compared to bona fide T cells [6, 9]. Together, this strongly suggests that myeloid phagocytes, unlike lymphocytes, generally make use of only a limited spectrum of the available combinatorial immune receptors.

Our finding that GM-CFUs derived from CD34^+^ cells express rearranged immunoglobulins strongly suggests that VDJ rearrangement takes place at an early phase during myeloid differentiation. It may occur even at an earlier stage as suggested by most recent work by others, which was published during the course of our study that reports Ig expression by CD34^+^ hematopoietic stem cells [37].

Ig expression was detected in ~3–5% of macrophages analyzed by immunofluorescent staining and single cell analysis. Interestingly, similar fractions of TCRαß expressing subpopulations of neutrophil granulocytes (5–8%) [6] and human CD14^+^ blood monocytes (~5%) [9] have recently been demonstrated. This reveals the existence of two small but distinct subpopulations in myeloid cells, which are able to rearrange the two variable immune receptor classes (Ig and TCR). The exact function of these variable immune receptors expressed by myeloid cells remains to be discovered.

However, it might be conceivable that macrophage-derived Ig are implicated in the anti-tumor immune response. The restricted repertoires, biased V- and J-chain usage and convergent recombination in the tumor macrophages may suggest that the Ig expressing macrophage population present in tumors engages in an antigen-directed immune response and the observed restricted Ig variants are the result of an adaptive response of macrophages to the tumor. By sequencing the Ig repertoires of a larger number of tumor samples preferably several samples of one tumor species we can establish a systematic overview of the macrophage Ig repertoires in the tumor milieu. Eventually, the distinct restriction of the TAM repertoires may prove to be a potential target for diagnostic applications. However, the general function of TAM remains controversial. In the future, the Ig expression in TAM can promote research in cancer immunity to uncover their role in tumor development and progress.

In line with this, we show for the first time the complete heavy and light chain sequences of a functional antibody expressed by a single macrophage isolated from the tumor microenvironment. The CD14^+^ macrophage was manually selected under the microscope and subjected to amplification and cloning of its recombined antibody genes. The identification of immunoglobulin heavy and light chain sequences from this tumor macrophage ultimately confirms that immunoglobulins are indeed produced by myeloid cells and not a result of residual B cells or any non-specific membranous binding of extracellular Ig. Although the CDR3 light chain sequence has been published in the context of West Nile Virus infection [54] and ocular adnexal marginal zone lymphoma [55], the heavy chain sequence is not listed in any database and the antigen specificity of this first myeloid antibody is thus far unknown. Current work is aimed to identify the antigen specificity of this first macrophage-derived antibody. With this information, it will be possible to begin addressing the biological function of the immunoglobulin-expressing macrophage subpopulation especially in the tumor microenvironment.

In conclusion, our results provide evidence for the existence of an immunoglobulin-based variable immune receptor system regularly generated by somatic genetic recombination in a subpopulation of human monocytes/ macrophages and also in TAM. These results add an all new aspect to our current understanding of the role of macrophages in immunity especially in the context of tumor host-defense.

## Supporting information

S1 Fig Immunoglobulin heavy chain gene expression in monocytes and *in vitro* differentiated macrophages. (PDF)

S2 Fig Detection of immunoglobulins in macrophages by immunoblot. (PDF)

S3 Fig Isotype control stainings of purified CD14^+^ monocytes. (PDF)

S4 Fig Analysis of the CD79b expression in PBMC by flow cytometry. (PDF)

S5 Fig Quantitative length variant analysis of the antigen-binding CDR3 region of Donor 3 and 4. (PDF)

S6 Fig Length variant analysis of the murine IgM V heavy chain antigen-binding CDR3 region. (PDF)

S7 Fig Quantitative length variant analysis of the antigen-binding CDR3 region of granulocyte/ macrophage progenitor colonies (CFU-GM). (PDF)

S8 Fig CDR3 Diversity index for immunoglobulin heavy (A) and light (B) chains.
(PDF)

S9 Fig Diversity index for VH segments of NGS sequences. (PDF)

S10 Fig B cell Ig heavy chain repertoire transcriptome profile assessed by ultra-deep sequencing. (PDF)

S11 Fig Immunoglobulin V/J gene usage. (PDF)

S1 Table List of CDR3 protein sequences and their expression frequencies in the analyzed cell samples. (PDF)

S2 Table Shared IgG, IgM and Igκ CDR3 protein sequences among different cell fractions. (PDF)

S3 Table List of all V(D)J combinations found in the sanger-sequenced cell fractions. (PDF)

S4 Table Shared V(D)J recombinations among different repertoires and cell types. (PDF)

S5 Table CDR3 sequences and the underlying VJ recombinations found in the NGS-sequenced samples TAM-4 and TAM-5. (PDF)

S6 Table Number of detected heavy and light chain sequences in single TAM isolated from different tumor samples. (PDF)

## References

[1] Chávez-Galán L, Olleros ML, Vesin D, Garcia I. Much more than M1 and M2 macrophages, there are also CD169+ and TCR+ macrophages. Front Immunol. 2015;6: 263 10.3389/fimmu.2015.00263 26074923 PMC4443739

[2] Janeway C.A. Jr., Medzhitov R. Innate immune recognition. Annu Rev Immunol. 2002;20: 197–216. 10.1146/annurev.immunol.20.083001.084359 11861602

[3] Hirano M, Das S, Guo P, Cooper MD. The evolution of adaptive immunity in vertebrates. Adv Immunol 2011;109: 125–157. 10.1016/B978-0-12-387664-5.00004-2 21569914

[4] Kaminski WE, Beham AW, Puellmann K. Extralymphocytic flexible immune recognition: a new angle on inflammation and aging. Aging Dis. 2012;3: 404–413. 23185720 PMC3501395

[5] Kaminski WE, Beham AW, Kzhyshkowska J, Gratchev A, Puellmann K. On the horizon: Flexible immune recognition outside lymphocytes. Immunobiol. 2013;218: 418–426. 10.1016/j.imbio.2012.05.024 22749215

[6] Puellmann K, Kaminski WE, Vogel M, Nebe CT, Schroeder J, Wolf H, et al http://www.ncbi.nlm.nih.gov/pubmed?term=Beham AW%5BAuthor%5D&cauthor=true&cauthor_uid=16983085. From the cover: A variable immunoreceptor in a subpopulation of human neutrophils. Proc Natl Acad Sci USA. 2006:103: 14441–14446. 10.1073/pnas.0603406103 16983085 PMC1599981

[7] Puellmann K, Beham AW, Kaminski WE. Cytokine storm and an anti-CD28 monoclonal antibody. N Engl J Med. 2006;355: 2592–2593. 10.1056/NEJMc062750 17167145

[8] Fuchs T, Puellmann K, Scharfenstein O, Eichner R, Stobe E, Becker A, et al The neutrophil variable TCR-like immune receptor is expressed across the entire human life span but repertoire diversity declines in old age. Biochem Biophys Res Commun. 2012;419:309–315. 10.1016/j.bbrc.2012.02.017 22342716

[9] Beham AW, Puellmann K, Laird R, Fuchs T, Streich R, Breysach C, et al A TNF-regulated recombinatorial macrophage immune receptor implicated in granuloma formation in tuberculosis. PloS Pathog. 2011;7: e1002375 10.1371/journal.ppat.1002375 22114556 PMC3219713

[10] Fuchs T, Puellmann K, Hahn M, Dollt C, Pechlivanidou I, Ovsiy I, et al A second combinatorial immune receptor in monocytes/macrophages is based on the TCRγδ. Immunobiol. 2013;218: 960–968. 10.1016/j.imbio.2012.11.005 23312956

[11] Legrand F, Driss V, Woerly G, Loiseau S, Hermann E, Fournié JJ, et al http://www.ncbi.nlm.nih.gov/pubmed?term=Héliot L%5BAuthor%5D&cauthor=true&cauthor_uid=19536290http://www.ncbi.nlm.nih.gov/pubmed?term=Mattot V%5BAuthor%5D&cauthor=true&cauthor_uid=19536290http://www.ncbi.nlm.nih.gov/pubmed?term=Soncin F%5BAuthor%5D&cauthor=true&cauthor_uid=19536290. A functional gammadelta TCR/CD3 complex distinct from gammadelta T cells is expressed by human eosinophils. PloS One. 2009;4: e5926 10.1371/journal.pone.0005926 19536290 PMC2693924

[12] Fuchs T, Puellmann K, Schneider S, Kruth J, Schulze TJ, Neumaier M, et al An autoimmune double attack. Lancet. 2012B;379(9823): 1364. 22483032 10.1016/S0140-6736(11)61939-9

[13] Lakschevitz FS, Aboodi GM, Glogauer M. Oral neutrophils display a site-specific phenotype characterized by expression ofT-cell receptor. J Periodontol. 2013;84: 1493–503. 10.1902/jop.2012.120477 23205919

[14] Fuchs T, Puellmann K, Emmert A, Fleig J, Oniga S, Laird R, et al The macrophage-TCRαβ is a cholesterol-responsive combinatorial immune receptor and implicated in atherosclerosis. Biochem Biophys Res Commun. 2015,456: 59–65. 10.1016/j.bbrc.2014.11.034 25446098

[15] Fuchs T, Hahn M, Riabov V, Yin S, Kzhyshkowska J, Busch S, et al A combinatorial αβ T cell receptor expressed by macrophages in the tumor microenvironment. Immunobiol. 2017;222: 39–44. 10.1016/j.imbio.2015.09.022 26494401

[16] Huang J, Sun X, Gong X, He Z, Chen L, Qiu X, et al Rearrangement and expression of the immunoglobulin mu-chain gene in human myeloid cells. Cell Mol Immunol 2014;11:94–104. 10.1038/cmi.2013.45 24141767 PMC4002143

[17] Wang C, Xia M, Sun X, He Z, Hu F, Chen L, et al IGK with conserved IGKV/IGKJ repertoire is expressed in acute myeloid leukemia and promotes leukemic cell migration. Oncotarget 2015;6: 39062–39072. doi: 10.18632/oncotarget.5393 26429876 10.18632/oncotarget.5393 PMC4770757

[18] Mantovani A, Sozzani S, Locati M, Allavena P, Sica A. Macrophage polarization: Tumor-associated macrophages as a paradigm for polarized M2 mononuclear phagocytes. Trends Immunol. 2002;23: 549–555. 12401408 10.1016/s1471-4906(02)02302-5

[19] Mantovani A, Schioppa T, Porta C, Allavena P, Sica A. Role of tumor-associated macrophages in tumor progression and invasion. Cancer Metastasis Rev. 2006;25, 315–322. 10.1007/s10555-006-9001-7 16967326

[20] Hagemann T, Robinson SC, Schulz M, Trümper L, Balkwill FR, Binder C. Enhanced invasiveness of breast cancer cell lines upon co-cultivation with macrophages is due to TNF-α dependent up-regulation of matrix metalloproteases. Carcinogenesis. 2004;25: 1543–1549. 10.1093/carcin/bgh146 15044327

[21] van der Bij GJ, Oosterling SJ, Meijer S, Beelen RHJ, van Egmond M. The role of macrophages in tumor development. Cell Oncol. 2005;27: 203–213. 10.1155/2005/719412 16308469 PMC4617500

[22] Tanaka Y, Kobayashi H, Suzuki M, Kanayama N, Suzuki M, Terao T. Thymidine phosphorylase expression in tumor-infiltrating macrophages may be correlated with poor prognosis in uterine endometrial cancer. Hum Pathol. 2002;33:1105–1113. 10.1053/hupa.2002.129203 12454815

[23] Noy R and Pollard JW. Tumor-Associated Macrophages: From mechanisms to therapy. Immunity. 2014;41: 49–61. 10.1016/j.immuni.2014.06.010 25035953 PMC4137410

[24] Bingle L, Brown NJ, Lewis CE. The role of tumour-associated macrophages in tumour progression: implications for new anticancer therapies. J Pathol. 2002;196: 254–265. 10.1002/path.1027 11857487

[25] Hanada T, Nakagawa M, Emoto A, Nomura T, Nasu N, Nomura Y. Prognostic value of tumor-associated macrophage count in human bladder cancer. Int J Urol. 2000;7: 263–269. 10910229 10.1046/j.1442-2042.2000.00190.x

[26] Kaminski WE, Lindahl P, Lin N, Broudy V, Crosby J, Hellström M, et al Basis of hematopoietic defects in platelet-derived growth factor (PDGF)-B and PDGF beta-receptor null mice. Blood 2001;97: 1990–1998. 11264163 10.1182/blood.v97.7.1990

[27] Van Dongen JJ, Langerak AW, Brüggemann M, Evans PA, Hummel M, Lavender FL, et al Design and standardization of PCR primers and protocols for detection of clonal immunoglobulin and T-cell receptor gene recombinations in suspect lymphoproliferations: report of the BIOMED-2 Concerted Action BMH4-CT98-3936. Leukemia. 2003;17: 2257–2317. 10.1038/sj.leu.2403202 14671650

[28] Kim SM, Bhonsle L, Besgen P, Nickel J, Backes A, Held K, et al Analysis of the paired TCR a- and ß-chains of single human T cells. PLoS One. 2012;7: 1–12. 10.1371/journal.pone.0037338 PMC3359365 22649519

[29] Tamura K, Peterson D, Peterson N, Stecher G, Nei M, Kumar S. MEGA5: Molecular Evolutionary Genetics Analysis Using Maximum Likelihood, Evolutionary Distance, and Maximum Parsimony Methods. Mol Biol Evol. 2011;28: 2731–2739. 10.1093/molbev/msr121 21546353 PMC3203626

[30] Retter I, Althaus HH, Münch R, Müller W. VBASE2, an integrative V gene database. Nucleic Acids Res. 2005;33: 671–674. 10.1093/nar/gki088 PMC540042 15608286

[31] Han J, Swan DC, Smith SJ, Lum SH, Sefers SE, Unger ER, et al Simultaneous Amplification and Identification of 25 Human Papillomavirus Types with Templex Technology. J Clin Microbiol.2006;44: 4157–4162. 10.1128/JCM.01762-06 17005760 PMC1698316

[32] Wang C, Sanders CM, Yang Q, Schroeder HWJ, Wang E, Babrzadeh F, et al High throughput sequencing reveals a complex pattern of dynamic interrelationships among human T cell subsets. Proc Natl Acad Sci U S A. 2010;107: 1518–1523. 10.1073/pnas.0913939107 20080641 PMC2824416

[33] Hutcheson K. A test for comparing diversities based on the Shannon formula. J Theor Biol. 1970;29: 151–154. 5493290 10.1016/0022-5193(70)90124-4

[34] Jost L. Entropy and diversity. Oikos. 2006;113: 363–375.

[35] Patton DT, Plumb AW, Abraham N. The Survival and Differentiation of Pro-B and Pre-B Cells in the Bone Marrow Is Dependent on IL-7Rα Tyr449. J Immunol. 2014;193: 3446–3455. 10.4049/jimmunol.1302925 25143441

[36] Goetz CA, Harmon IR, O’Neil JJ, Burchill MA, Farrar MA. STAT5 Activation Underlies IL7 Receptor-Dependent B Cell Development. J Immunol. 2004;172: 4770–4778. 15067053 10.4049/jimmunol.172.8.4770

[37] Liu J, Xia M, Wang P, Wang C, Geng Z, Cameron Yin C, et al Immunoglobulin gene expression in umbilical cord blood-derived CD34+ hematopoietic stem/progenitor cells. Gene. 2016;575: 108–117. 10.1016/j.gene.2015.08.046 26364572

[38] Jackson KJL, Kid MJ, Wang Y, Collins AM. The shape of the lymphocyte receptor repertoire: lessons from the B cell receptor. Front immunol.2013; 4:263 10.3389/fimmu.2013.00263 24032032 PMC3759170

[39] Yang Y, Wang C, Yang Q, Kantor AB, Chu H, Ghosn EEB, et al Distinct mechanisms define murine B cell lineage immunoglobulin heavy chain (IgH) repertoires. eLife 2015;4: e09083 10.7554/eLife.09083 26422511 PMC4714975

[40] Zheng J, Huang J, Mao Y, Liu S, Sun X, Zhu X, et al Immunoglobulin gene transcripts have distinct VHDJH recombination characteristics in human epithelial cancer cells. J Biol Chem. 2009;284:13610–13619. 10.1074/jbc.M809524200 19293154 PMC2679462

[41] Liu HD, Zheng H, Li M, Hu DS, Tang M, Cao Y. Upregulated expression of kappa light chain by Epstein-Barr virus encoded latent membrane protein 1 in nasopharyngeal carcinoma cells via NF-kappaB and AP-1 pathways. Cell Signal. 2007;19: 419–427. 10.1016/j.cellsig.2006.07.012 16979873

[42] Qiu X, Sun X, He Z, Huang J, Hu F, Chen L, et al Immunoglobulin gamma heavy chain gene with somatic hypermutation is frequently expressed in acute myeloid leukemia. Leukemia. 2013; 27: 92–99. 10.1038/leu.2012.184 22772058

[43] Deng YQ, Zheng J, Li GH, Zhu XH, Zhang P, Huang J, et al Immunoglobulin expression in colon cancer cell line HT-29 and its biological activities. Zhonghua Zhong Liu Za Zhi. 2006;28: 88–91. 16750008

[44] Kimoto Y. Expression of heavy-chain constant region of immunoglobulin and T-cell receptor gene transcripts in human non-hematopoietic tumor cell lines. Genes Chromosomes Cancer. 1998;22: 83–86. 9591639 10.1002/(sici)1098-2264(1998)22:1<83::aid-gcc12>3.0.co;2-o

[45] Babbage G, Ottensmeier CH, Blaydes J, Stevenson FK, Sahota SS. Immunoglobulin heavy chain locus events and expression of activation-induced cytidine deaminase in epithelial breast cancer cell lines. Cancer Res. 2006;66: 3996–4000. 10.1158/0008-5472.CAN-05-3704 16618718

[46] Huang J, Sun X, Mao Y, Zhu X, Zhang P, Zhang L, et al Expression of immunoglobulin gene with classical V-(D)-J rearrangement in mouse brain neurons. Int J Biochem Cell Biol. 2008;40: 1604–1615. 10.1016/j.biocel.2007.12.004 18243769

[47] Shao W, Zhang C, Liu E, Zhang L, Ma J, Zhu Z, et al Identification of liver epithelial cell-derived Ig expression in μ chain-deficient mice. Scientific Reports. 2016;6: 23669 10.1038/srep23669 27020674 PMC4810322

[48] Jiang D, Ge J, Liao Q, Ma J, Liu Y, Huang J, et al IgG and IgA with potential microbial-binding activity are expressed by normal human skin epidermal cells. Int J Mol Sci. 2015;16: 2574–2590. 10.3390/ijms16022574 25625513 PMC4346852

[49] Zhao Y, Liu Y, Chen Z, Korteweg C, Gu J. Immunoglobulin g (IgG) expression in human umbilical cord endothelial cells. J Histochem Cytochem. 2011;59: 474–488. 10.1369/0022155411400871 21430258 PMC3201178

[50] Hayakawa K, Hardy RR, Parks DR, Herzenberg LA. The “Ly-1 B” cell subpopulation in normal immunodefective, and autoimmune mice. J Exp Med. 1983;157: 202–218. 6600267 10.1084/jem.157.1.202 PMC2186909

[51] Wang S, Xia P, Chen Y, Huang G, Xiong Z, Liu J, et al Natural Killer-like B Cells Prime Innate Lymphocytes against Microbial Infection. Immunity. 2016;45: 131–144. 10.1016/j.immuni.2016.06.019 27421702

[52] Popi A. B-1 phagocytes: the myeloid face of B-1 cells. Ann NY Acad Sci. 2015;1362: 86–97. 10.1111/nyas.12814 26149496

[53] Griffin DO and Thomas L. A small CD11b+ human B1 cell subpopulation stimulates T cells and is expanded in lupus. J Exp Med. 2011;208: 2591–2598. 10.1084/jem.20110978 22110167 PMC3244038

[54] Throsby M, Geuijen C, Goudsmit J, Bakker AQ, Korimbocus J, Kramer RA, et al Isolation and Characterization of Human Monoclonal Antibodies from Individuals Infected with West Nile Virus. J Virol. 2006;80: 6982–6992. 10.1128/JVI.00551-06 16809304 PMC1489037

[55] Zhu D, Lossos C, Chapman-Fredricks JR, Lossos IS. Biased Immunoglobulin Light Chain Use in the *Chlamydophila psittaci* Negative Ocular Adnexal Marginal Zone Lymphomas. Am J Hematol. 2013;88: 379–384. 10.1002/ajh.23416 23418012 PMC3644856

