# Supplementary material for: Expression of combinatorial immunoglobulins in macrophages in the tumor microenvironment
Source: PLoS One. 2018 Sep 21;13(9):e0204108. doi: 10.1371/journal.pone.0204108 (PMC6150476; doi:10.1371/journal.pone.0204108)
Supplement: S1 Fig — (PDF) [file pone.0204108.s001.pdf]

Figure S1

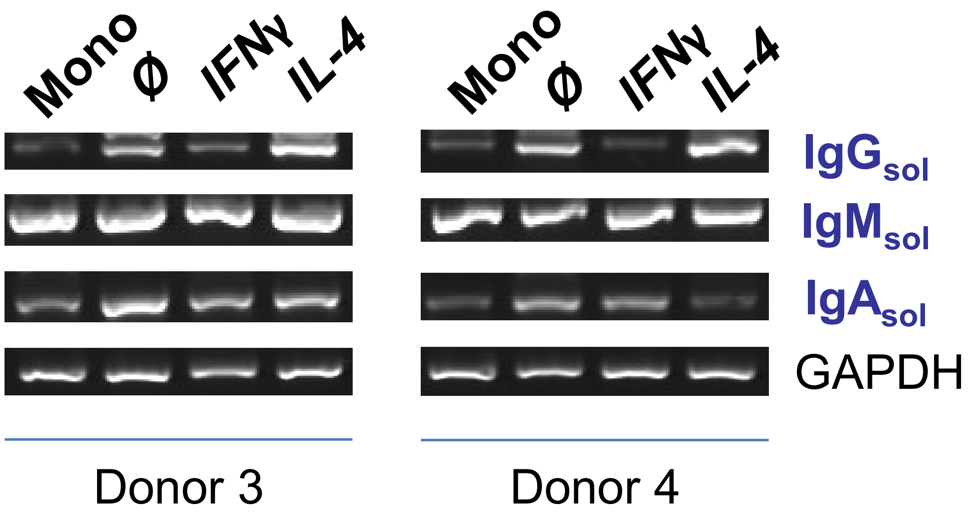

Immunglobulin heavy chain gene expression in monocytes and *in vitro* differentiated macrophages. RT-PCR profiling for the immunoglobulin heavy chain genes (IgG, IgM, IgA) is shown for CD14+ monocytes and in vitro differentiated macrophages (without any stimulus (Ø), IFNγ or IL-4, respectively from two representative healthy individuals (Donor 3 and 4). GAPDH is shown as a house-keeping control. IFNγ: Interferon γ; IL-4: Interleukin-4; GAPDH: Glycerinaldehyd-3-phosphat-dehydrogenase.
