# Supplementary material for: Expression of combinatorial immunoglobulins in macrophages in the tumor microenvironment
Source: PLoS One. 2018 Sep 21;13(9):e0204108. doi: 10.1371/journal.pone.0204108 (PMC6150476; doi:10.1371/journal.pone.0204108)
Supplement: S2 Fig — (PDF) [file pone.0204108.s002.pdf]

Figure S2

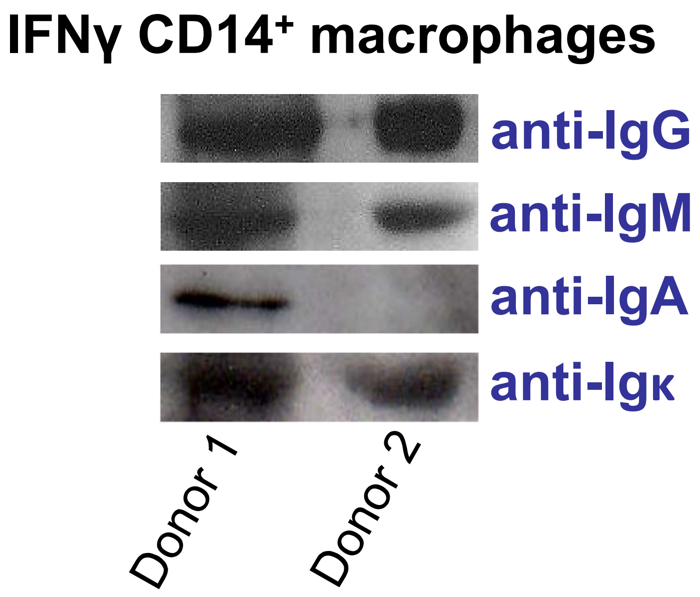

Detection of immunoglobulins in macrophages by immunoblot. Whole cell lysates of IFNγ-activated CD14$^+$ macrophages from donors 1 and 2 were separated under reducing conditions. Antibodies against IgM, IgG, IgA and Igκ, respectively, were used.
