# Supplementary material for: Expression of combinatorial immunoglobulins in macrophages in the tumor microenvironment
Source: PLoS One. 2018 Sep 21;13(9):e0204108. doi: 10.1371/journal.pone.0204108 (PMC6150476; doi:10.1371/journal.pone.0204108)
Supplement: S3 Fig — (PDF) [file pone.0204108.s003.pdf]

Figure S3

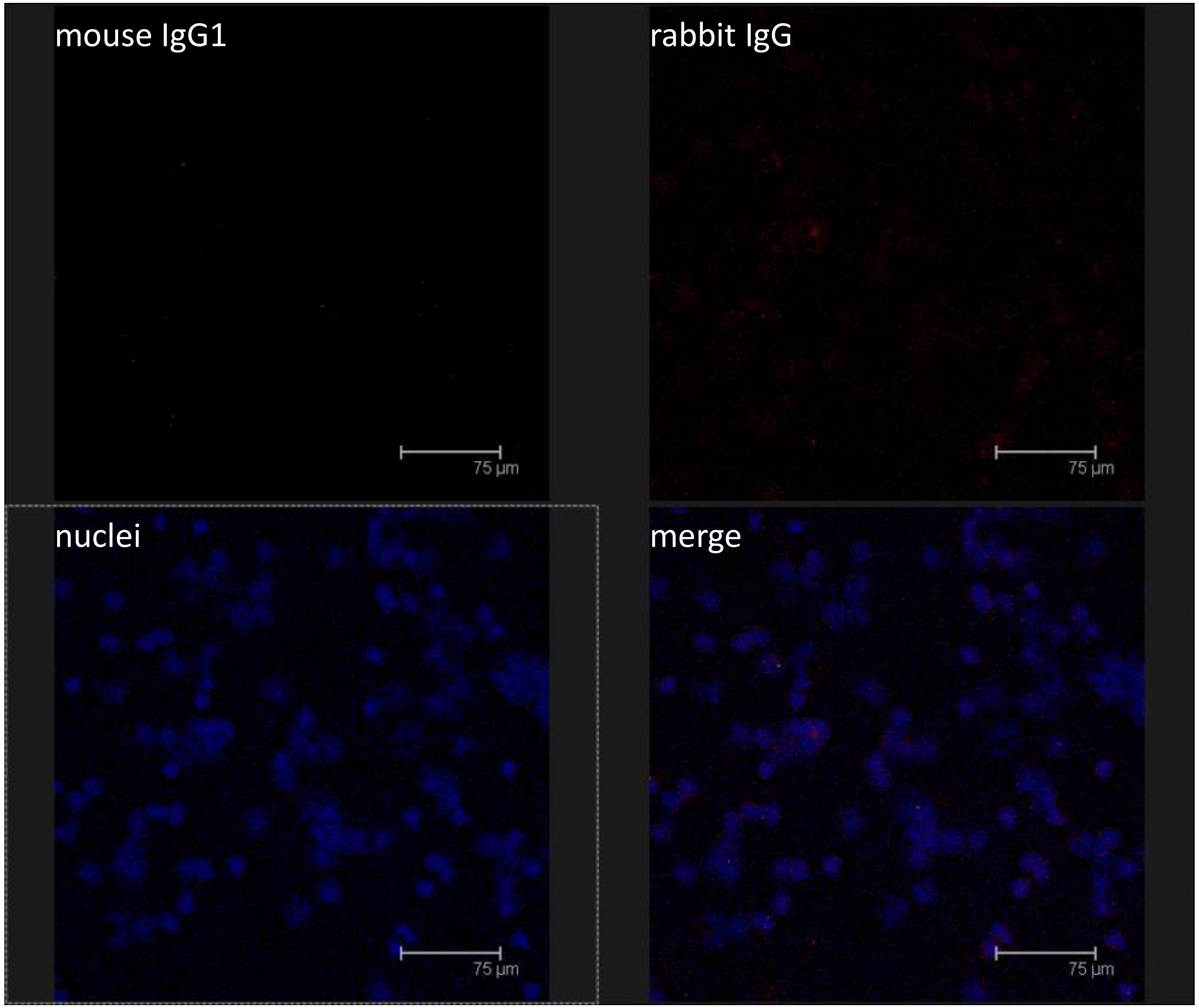

Isotype control stainings of purified CD14⁺ monocytes. Note the absence of immunostaining, when isotype-matched control antibodies were used in place of the anti-TCRaβ (mouse IgG1) and the anti-IgM (rabbit IgG) primary antibodies. Nuclei (blue) are counterstained with DRAQ5.
