# Supplementary material for: Expression of combinatorial immunoglobulins in macrophages in the tumor microenvironment
Source: PLoS One. 2018 Sep 21;13(9):e0204108. doi: 10.1371/journal.pone.0204108 (PMC6150476; doi:10.1371/journal.pone.0204108)
Supplement: S4 Fig — (PDF) [file pone.0204108.s004.pdf]

Figure S4

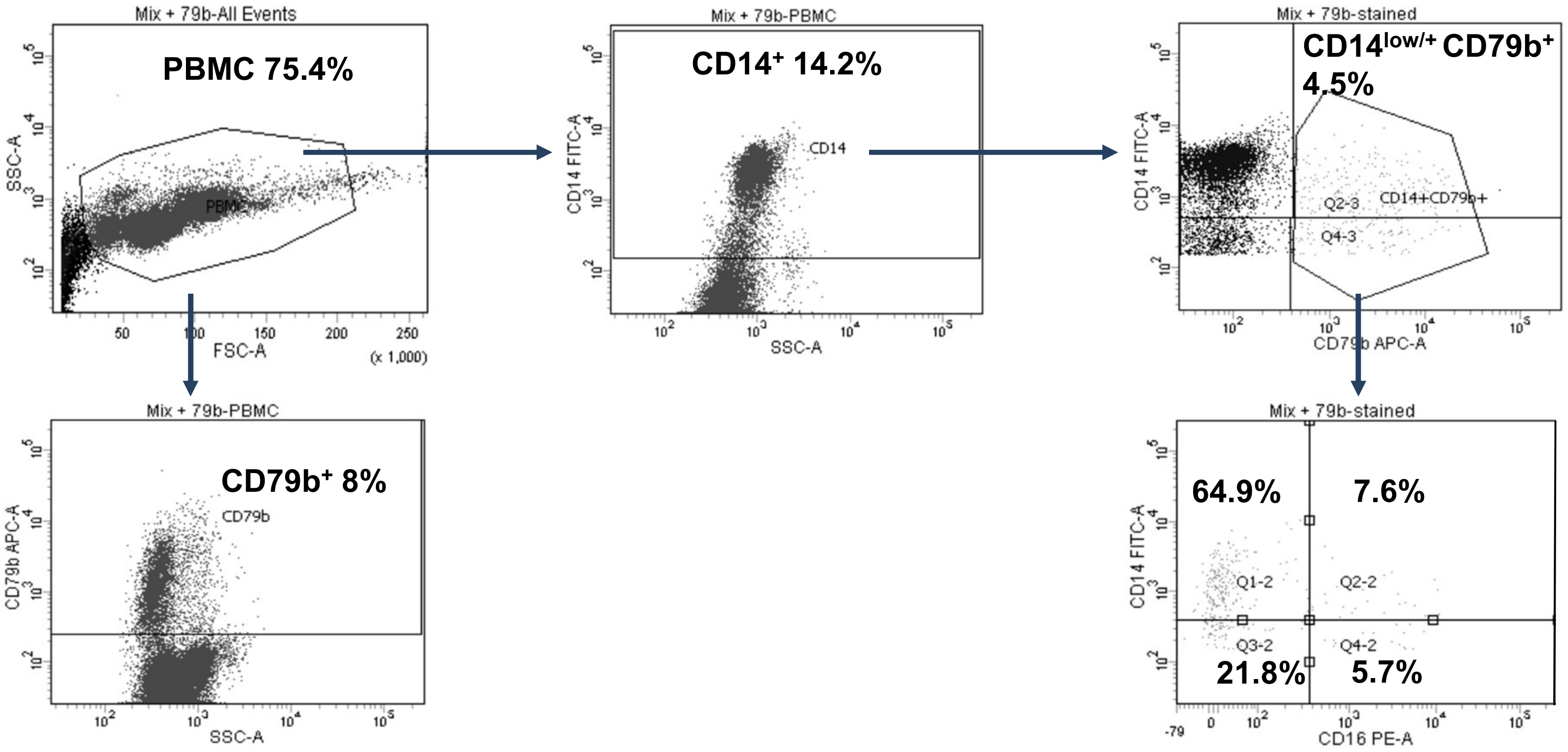

Analysis of the CD79b expression in PBMC by flow cytometry. Flow cytometry of freshly isolated PBMC representatively shown for one individual demonstrates the expression of CD79b in CD14$^{+/low}$ monocytes. Note the diverse distribution of the CD79b expression in the three monocyte subgroups CD14^{+}/CD16^{-} (classical monocytes; 64.9%); CD14^{+}/CD16^{+} (intermediate monocytes; 7.6%) and CD14^{low}/CD16^{+} (non-classical monocytes; 5.7%). Using this surrogate marker for immunoglobulin expression suggests the classical monocytes as the main immunoglobulin producing subgroup.
