# Supplementary material for: Expression of combinatorial immunoglobulins in macrophages in the tumor microenvironment
Source: PLoS One. 2018 Sep 21;13(9):e0204108. doi: 10.1371/journal.pone.0204108 (PMC6150476; doi:10.1371/journal.pone.0204108)
Supplement: S5 Fig — (PDF) [file pone.0204108.s005.pdf]

Figure S5A

Donor 3

IgM **IgG** **IgA**

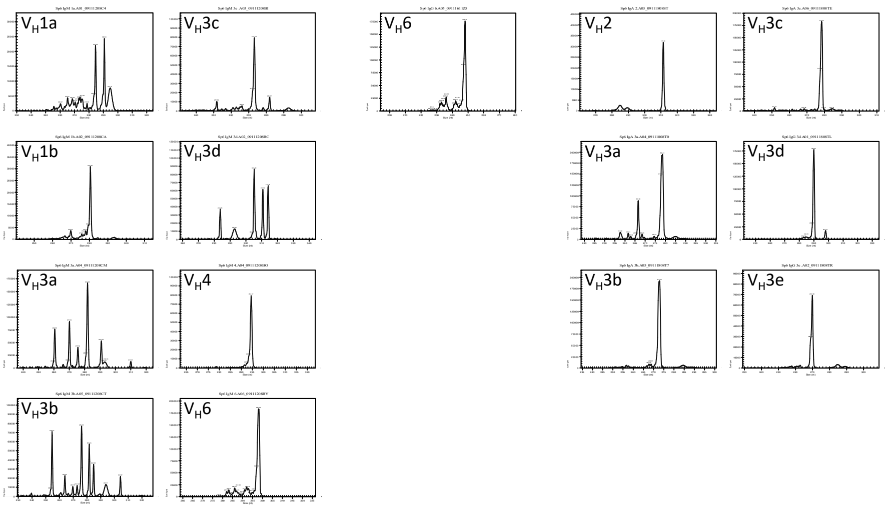

Figure S5B

Donor 4

IgM **IgG** **IgA**

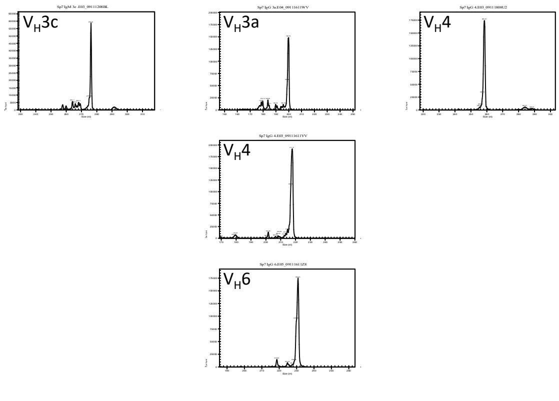

Quantitative length variant analysis of the antigen-binding CDR3 region of Donor 3 and 4. Length variant analysis of the antigen-binding complementarity determining region 3 (CDR3) demonstrates the constitutive expression of individual-specific IgM, IgG and IgA variable heavy chain repertoires, respectively, by IFNγ macrophages from two healthy donors (donor 3 and 4). V_H – $C_{M/G/A}$ specific cDNA segments were amplified by RT-PCR and separated by capillary electrophoresis ("CDR3 spectratyping"). The detailed CDR3 length spectratypes for each expressed V chain are shown. Peak heights are indicated as fluorescence units.
