# Supplementary material for: Expression of combinatorial immunoglobulins in macrophages in the tumor microenvironment
Source: PLoS One. 2018 Sep 21;13(9):e0204108. doi: 10.1371/journal.pone.0204108 (PMC6150476; doi:10.1371/journal.pone.0204108)
Supplement: S6 Fig — (PDF) [file pone.0204108.s006.pdf]

Figure S6

CD11b$^+$ bone marrow

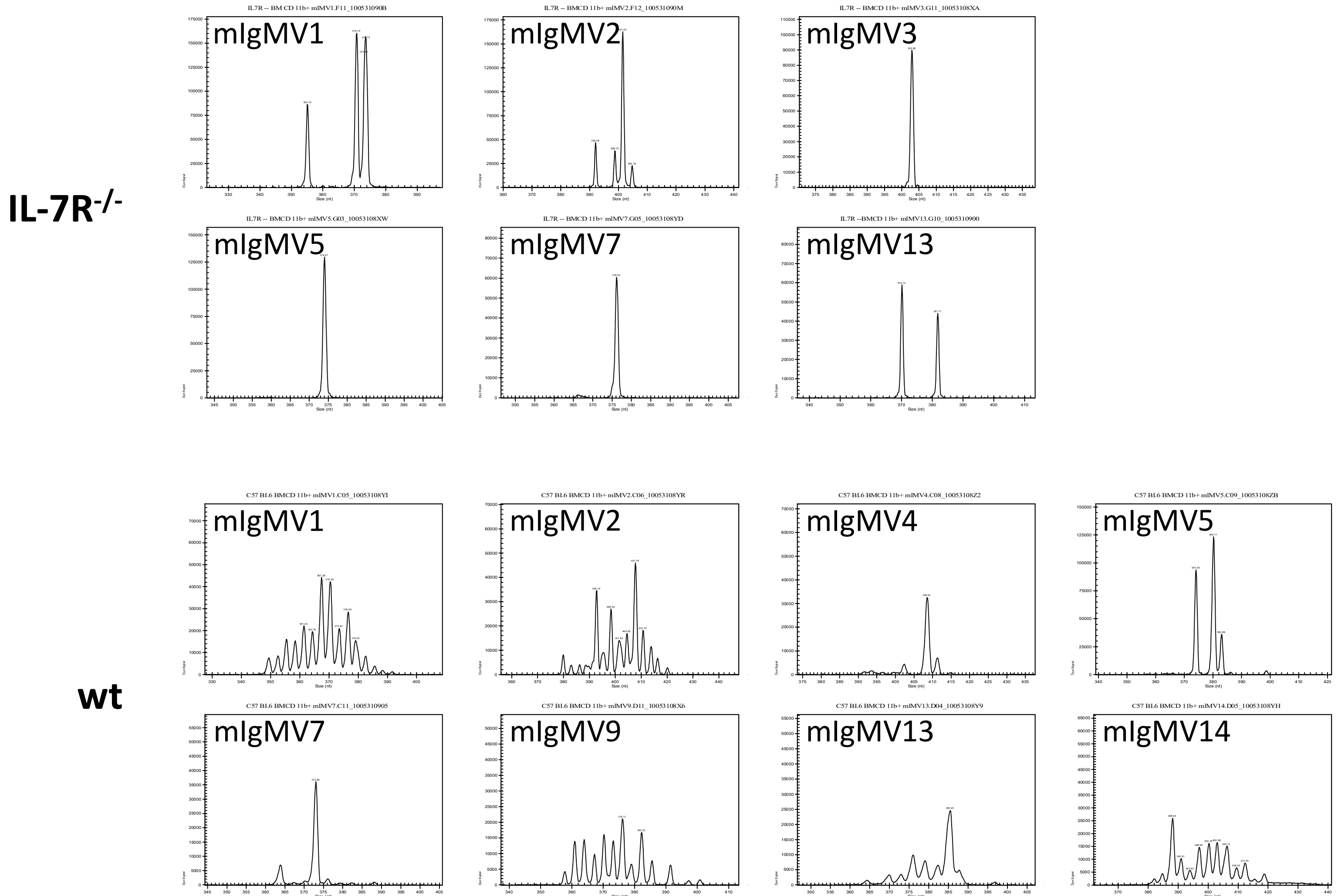

Length variant analysis of the murine IgM V heavy chain antigen-binding CDR3 region. Length variant analysis of the antigen-binding complementarity determining region 3 (CDR3) demonstrates the constitutive expression of mIgM variable heavy chain repertoires by bone marrow CD11b$^+$ macrophages from wildtype mice (wt) and mice lacking lymphocytes (IL-7 receptor knockout mice, IL-7R$^{-/-}$). $V_H – C_M$ specific cDNA segments were amplified by RT-PCR and separated by capillary electrophoresis ("CDR3 spectratyping"). The detailed CDR3 length spectratypes for each expressed V chain are shown. Peak heights are indicated as fluorescence units.
