# Supplementary material for: Expression of combinatorial immunoglobulins in macrophages in the tumor microenvironment
Source: PLoS One. 2018 Sep 21;13(9):e0204108. doi: 10.1371/journal.pone.0204108 (PMC6150476; doi:10.1371/journal.pone.0204108)
Supplement: S7 Fig — (PDF) [file pone.0204108.s007.pdf]

Figure S7A

CFU-GM 1

CFU-GM 2

CFU-GM 5

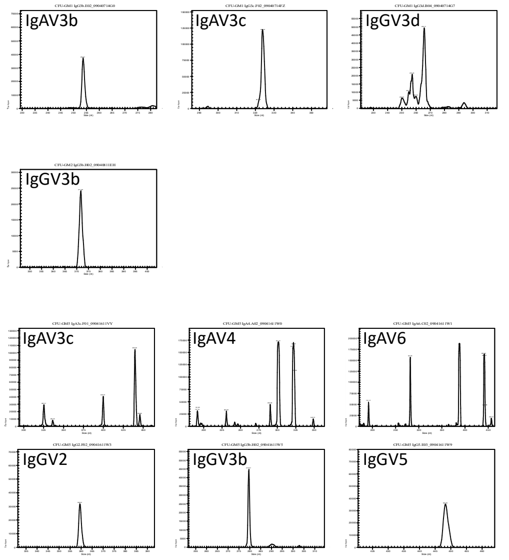

Figure S7B

CFU-GM 6

CFU-GM 7

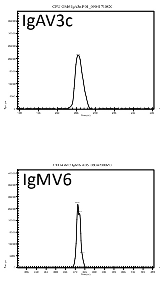

Quantitative length variant analysis of the antigen-binding CDR3 region of granulocyte/ macrophage progenitor colonies (CFU-GM). Length variant analysis of the antigen-binding complementarity determining region 3 (CDR3) shows the expression of IgM, IgG and IgA variable heavy chain repertoires, respectively, by seven granulocyte/ macrophage progenitor colonies obtained from CD34$^+$ progenitors of a healthy donor. V_H – $C_{M/G/A}$ specific cDNA segments were amplified by RT-PCR and separated by capillary electrophoresis ("CDR3 spectratyping"). The detailed CDR3 length spectratypes for each expressed V chain are shown. Peak heights are indicated as fluorescence units.
