# Supplementary material for: Expression of combinatorial immunoglobulins in macrophages in the tumor microenvironment
Source: PLoS One. 2018 Sep 21;13(9):e0204108. doi: 10.1371/journal.pone.0204108 (PMC6150476; doi:10.1371/journal.pone.0204108)
Supplement: S8 Fig — CDR3 Diversity index for immunoglobulin heavy (A) and light (B) chains. (PDF) [file pone.0204108.s008.pdf]

Figure 8A

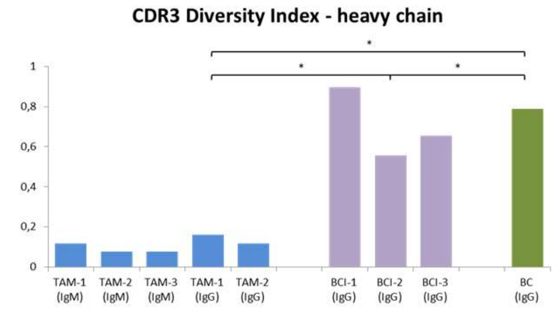

Figure 8B

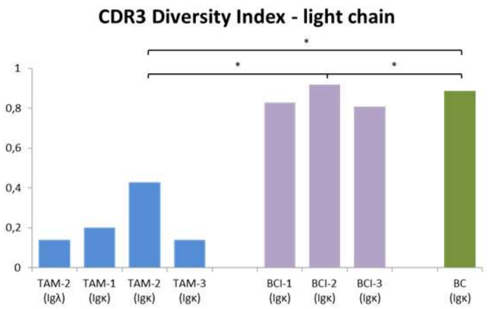

CDR3 Diversity Index for immunoglobulin heavy (A) and light (B) chains. CDR3 heavy **(A)** and light chain **(B)** repertoire diversity indices for immunoglobulins expressed by TAM obtained from three distinct cancer patients (TAM-1, TAM-2, TAM-3). Note that TAM express markedly less diverse repertoires relative to B cells from patients with inflammatory diseases (BCI-1, BCI-2, BCI-3) and normal B cells (BC). The diversity index indicates the ratio of distinct clonotypes to the total number of sequenced clones. *=p<0.05.
