# Supplementary material for: Expression of combinatorial immunoglobulins in macrophages in the tumor microenvironment
Source: PLoS One. 2018 Sep 21;13(9):e0204108. doi: 10.1371/journal.pone.0204108 (PMC6150476; doi:10.1371/journal.pone.0204108)
Supplement: S9 Fig — (PDF) [file pone.0204108.s009.pdf]

Figure S9

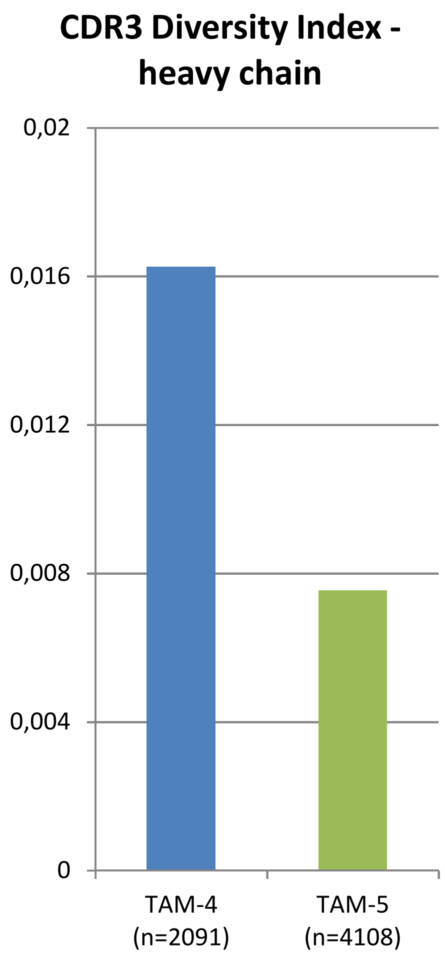

Diversity Index for V_H segments of NGS sequences. CDR3 heavy chain repertoire diversity indices for immunoglobulins expressed by NGS sequenced TAM obtained from two distinct cancer patients (TAM-4, TAM-5, TAM-3). The diversity index indicates the ratio of unique clonotypes to the total number of sequenced clones. The number of clones analyzed for each repertoire is shown below the columns.
