# Supplementary material for: Expression of combinatorial immunoglobulins in macrophages in the tumor microenvironment
Source: PLoS One. 2018 Sep 21;13(9):e0204108. doi: 10.1371/journal.pone.0204108 (PMC6150476; doi:10.1371/journal.pone.0204108)
Supplement: S10 Fig — (PDF) [file pone.0204108.s010.pdf]

Figure S10

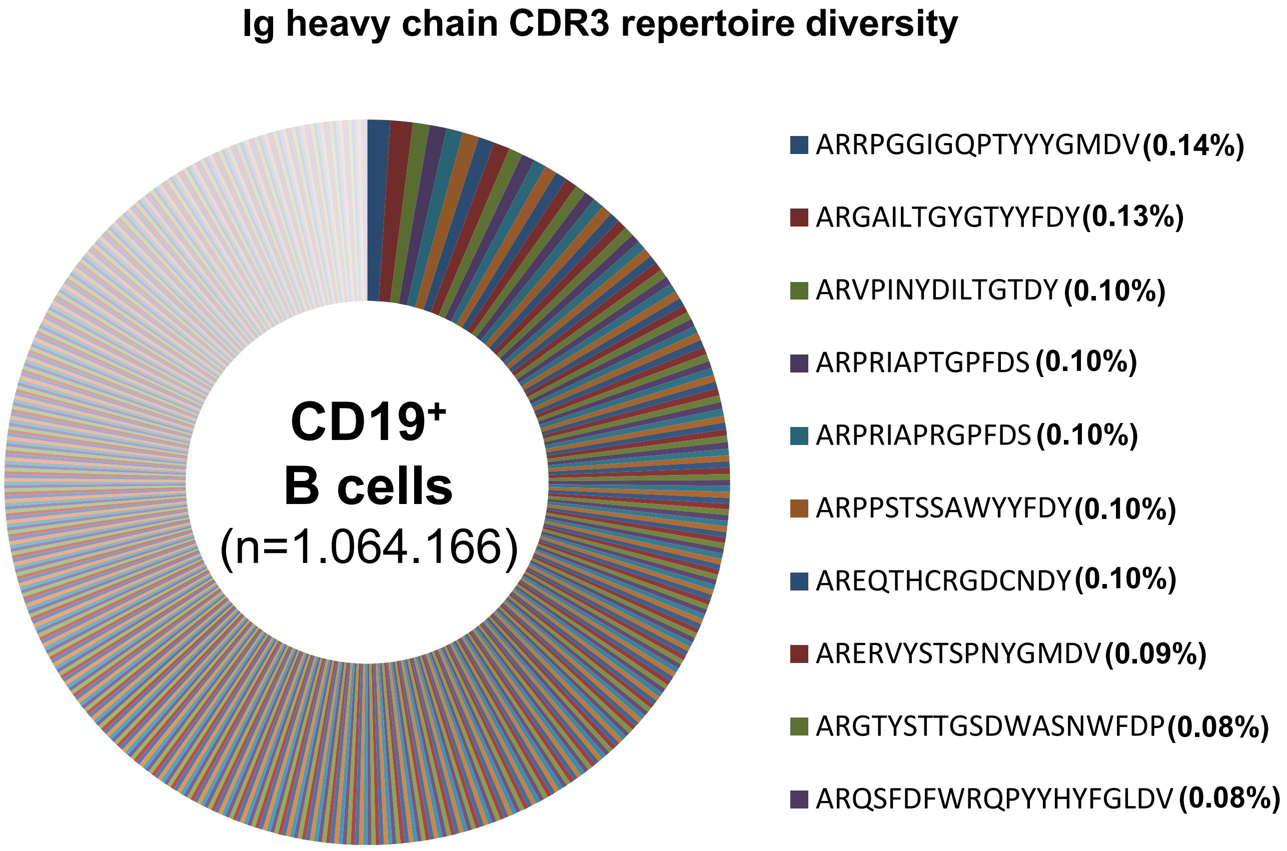

B cell Ig heavy chain repertoire transcriptome profile assessed by ultra-deep sequencing.
Shown is the Ig V_H CDR3 repertoires of CD19+ B cells isolated from a healthy individual as assessed by ultra-deep sequencing. The circle graph visualizes the relative abundance of the expressed Ig heavy chain CDR3 transcript variants. Each sector in a circle represents a rearranged Ig heavy chain transcript that encodes a unique CDR3 sequence. It is defined by a unique color and its area is proportional to the relative transcript frequency. The total numbers of identified Ig V_H CDR3 variants are indicated in the center. The 10 most frequently expressed Ig heavy chain CDR3 variants are shown in detail and the respective percentages are shown in parentheses.
