# Supplementary material for: Expression of combinatorial immunoglobulins in macrophages in the tumor microenvironment
Source: PLoS One. 2018 Sep 21;13(9):e0204108. doi: 10.1371/journal.pone.0204108 (PMC6150476; doi:10.1371/journal.pone.0204108)

Figure S11A

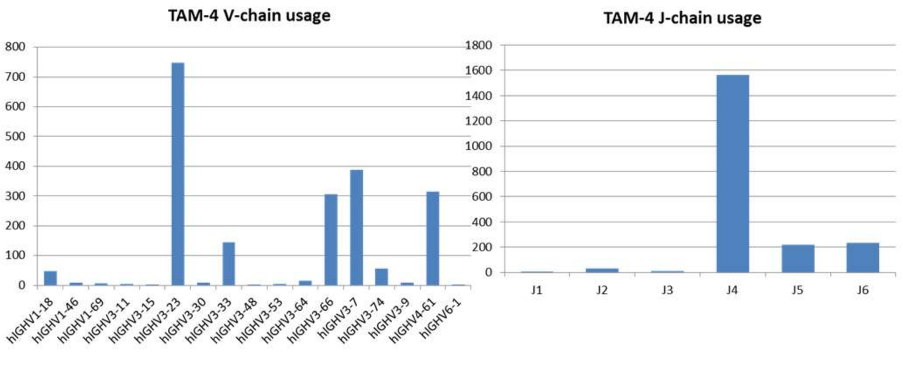

Immunoglobulin V/J gene usage. IgHV and IgHJ gene usage by TAM-4 **(A)** and TAM-5 **(B)**, respectively. The 2D-plots show the relative usage of the $IgHV_i$ (left) and $IgHJ_i$ (right) genes by TAM from each individual. X-axis: IgHV gene/IgHJ gene. Y-axis: number of used IgHV/IgHJ genes

Figure S11B

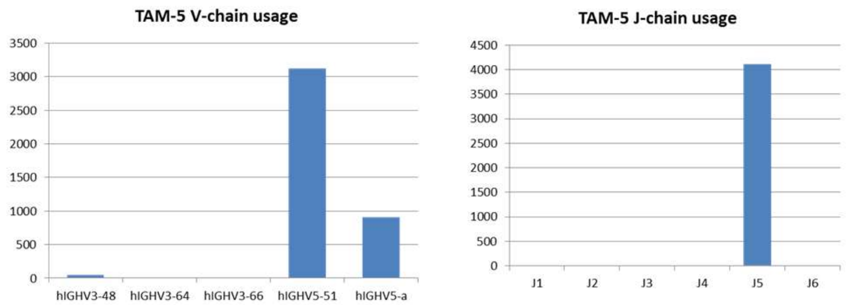

Supplement: S11 Fig — (PDF) [file pone.0204108.s011.pdf]
