# Supplementary material for: Expression of combinatorial immunoglobulins in macrophages in the tumor microenvironment
Source: PLoS One. 2018 Sep 21;13(9):e0204108. doi: 10.1371/journal.pone.0204108 (PMC6150476; doi:10.1371/journal.pone.0204108)
Supplement: S1 Table — (PDF) [file pone.0204108.s012.pdf]

Table S1: List of CDR3 protein sequences and their expression frequencies in the analyzed cell samples

	**IgM (n=34)**		**IgG (n=37)**		**Igκ (n=15)**			
**TAM-1**	ARIPAYCASTSCYEDGWGY	26 (76%)	ARGRGLRFLEWFIDY	18 (49%)	QQYYPYSGS	7 (47%)		
	ARPMVRGARDAFDI	5 (15%)	ARIPGSYFTGRYYFDY	7 (19%)	QQYDSSIT	6 (40%)		
	ASRIIGTINC	2 (6%)	ARGPSVSGYSYYFKGMDV	6 (16%)	LQYYSYPWT	2 (13%)		
	ARIPGSYFTGRYYFDY	1 (3%)	AKELSVEAAATDALDI	3 (8%)				
			ARGPSVSGYSFFYKGMDV	2 (5%)				
			ARGSGGTLITFGGVIPYFDY	1 (3%)				
	**IgM (n=39)**		**IgG (n=34)**		**Igκ (n=28)**		**Igλ (n=36)**	
**TAM-2**	ARVPINYDILTGTDY	37 (95%)	ARGSRIEVVDLWWPQEAAFDI	20 (59%)	QQYDNLPYT	5 (18%)	LLTYGGARV	28 (78%)
	ARVPINYDILTGTDC	1 (3%)	ASAGMNYYYGMDV	10 (29%)	QQYDNLSALT	5 (18%)	QVWDTSSDHRRV	5 (14%)
	ARVPINYDTLTGTDY	1 (3%)	AREHSRGLSFDY	2 (6%)	QHYVGSPGGTMYT	3 (11%)	LLAYGGARV	1 (3%)
			AKARREYYGVGNYFPE	2 (6%)	QQYGSSWWT	3 (11%)	QAWDSSTVV	1 (3%)
					QQYNSYLYT	3 (11%)	QVWDSSSDHHVV	1 (3%)
					QQYNKWPPLT	2 (7%)		
					QQYNSYPLT	2 (7%)		
					HHYGTSPYT	1 (4%)		
					MQALQTPA	1 (4%)		
					QQYESFPFT	1 (4%)		
					QQYNSYSLT	1 (4%)		
					QQYNSYSYT	1 (4%)		
	**IgM (n=51)**				**Igκ (n=36)**			
**TAM-3**	ARVPINYDILTGTDY	37 (73%)			QQYNTYPLT	19 (53%)		
	ARGYYGMDV	12 (24%)			QQYDNLPYT	10 (28%)		
	ARVPINYDIWTGTDY	1 (2%)			QQYDNLSALT	6 (17%)		
	AGVPINYDILTGTDY	1 (2%)			QQSYSTPRT	1 (3%)		
			**IgG (n=39)**		**Igκ (n=35)**			
**BCI-1**			ARDPYYYGSSTFDP	2 (5%)	KQYNAYPIT	2 (6%)		
			AREALPYDFWGDHYPRDSVEV	2 (5%)	QQSYSTPRT	2 (6%)		
			ARGRCGGDCLRRYYFDY	2 (5%)	QQSYSTPYT	2 (6%)		
			ARVIATVSTSGLDY	2 (5%)	QQSYSTSLT	2 (6%)		
			AALKVGGTRSFDY	1 (3%)	QQYDNLPRT	2 (6%)		
			AGVRGDSFILFVF	1 (3%)	QQYGNSPLT	2 (6%)		
			AKDLGHLVVVTASKIDR	1 (3%)	LQDYNYPRT	1 (3%)		
			AKDTAISGGEYGMDV	1 (3%)	LQHDNFPLT	1 (3%)		
			AKHTRGSVFLGTLDI	1 (3%)	LQHDNFPWT	1 (3%)		
			AKLTDY	1 (3%)	QQSYSTLT	1 (3%)		
			AKLVSN	1 (3%)	QQSYSTPRAT	1 (3%)		
			ARDFLYDFSAPMPKAFDS	1 (3%)	QQSYSTPWT	1 (3%)		
			ARDKGTSGGAFDI	1 (3%)	QQSYSTRWT	1 (3%)		
			ARDNFAGGFEGDYYYYYGLDV	1 (3%)	QQSYSTSWT	1 (3%)		
			AREALPYDFWGDYYPRDSVEV	1 (3%)	QQTYSFPHT	1 (3%)		
			AREGVNLLLSSDGAGARHYLDY	1 (3%)	QQYDNLIT	1 (3%)		
			AREKPHTLYFDY	1 (3%)	QQYDNLPGT	1 (3%)		
			AREYIWRFLRWFDP	1 (3%)	QQYDNLPLYT	1 (3%)		
			ARGKGSEYRDAFDM	1 (3%)	QQYDNLPPR	1 (3%)		
			ARGTRNNWINPGFDH	1 (3%)	QQYDNLSLLT	1 (3%)		
			ARGTRNNWISVGFDL	1 (3%)	QQYGSSPWT	1 (3%)		
			ARHAGPTYFDY	1 (3%)	QQYGSSSELT	1 (3%)		
			ARLYPRPGITETRRFDP	1 (3%)	QQYGTSPTT	1 (3%)		
			ARNLYGTGTFED	1 (3%)	QQYNSYSGT	1 (3%)		
			ARVLSIRTQCVSGPCPNAWFDP	1 (3%)	QQYNSYSPYT	1 (3%)		
			ARVSPMTTVTTFDY	1 (3%)	QQYNTYPLT	1 (3%)		
			ASGYPWGNCPEKSEDY	1 (3%)	QQYYSTPLT	1 (3%)		
			GRHAGPTYFDY	1 (3%)	QQYYSTYT	1 (3%)		
			IKGGSRSGWYGCGDF	1 (3%)	QQYYSYPLT	1 (3%)		
			SVDINPGGAAV	1 (3%)				
			VKDKSRGTYADAFDI	1 (3%)				
			VRGCTGDCVSGTDYYYGLDV	1 (3%)				
			VRSVGRGPDYWVVFGY	1 (3%)				
			VRTGGCRDGATAICLQH	1 (3%)				
			VTTSSPWLDVWDKGTPVTVSS	1 (3%)				
			**IgG (n=18)**		**Igκ (n=25)**			
**BCI-2**			ARQPSIVRLSNYLYYMDV	5 (28%)	QEYNSAPWT	2 (8%)		
			ARGQGSSYYGHFDY	3 (17%)	QQYSVNSA	2 (8%)		
			ARHSSKGVVVPSRPRWFLDL	2 (11%)	LQDYTYPLT	1 (4%)		
			ARQAGSSPLEH	2 (11%)	LQYERYWT	1 (4%)		
			AKGGYYHGGGYWFDP	1 (6%)	QHYDNLPP	1 (4%)		
			ARHGLAVAGTRWFDP	1 (6%)	QHYNGYPYT	1 (4%)		
			ARTYGSSGWYVFDY	1 (6%)	QKYNSVPT	1 (4%)		
			TRRSSRELWPPV	1 (6%)	QQANSFPLT	1 (4%)		
			TTELWLVPEMLY	1 (6%)	QQNYRTLT	1 (4%)		
			VALRYCSGSGCNWFDP	1 (6%)	QQSYSDPLT	1 (4%)		
					QQSYSSRT	1 (4%)		
					QQSYSTPLT	1 (4%)		
					QQTYSTPWT	1 (4%)		
					QQYDSHPVA	1 (4%)		
					QQYGSSPPLT	1 (4%)		
					QQYGSSPPS	1 (4%)		
					QQYGSSPRT	1 (4%)		
					QQYKSDYPVT	1 (4%)		
					QQYNSYPWT	1 (4%)		
					QQYNSYPYT	1 (4%)		
					QQYSRYSRK	1 (4%)		
					QQYSSSLWT	1 (4%)		
					QRYDNFPPFT	1 (4%)		
			**IgG (n=32)**		**Igκ (n=26)**			
			ARDGAVGRRGGYYLDY	5 (16%)	QQSYSTPYT	3 (12%)		
			ARAVNGVFDY	3 (9%)	QQRSNWPLT	2 (8%)		
			VKAGLWGSAQYYYYAMDV	3 (9%)	QQSYSTLLT	2 (8%)		
			AKDDVGVGNWFTP	2 (6%)	QQYDNLPQIT	2 (8%)		
			ARDRRESSAWWEHENDAFDV	2 (6%)	HQTYRIPFT	1 (4%)		

BCI-3		ARKYDYYHGISGYYYFDY	2 (6%)	LEDYDYPWT	1 (4%)	
		AGGRGYTYGYAY	1 (3%)	MQGTYWPWT	1 (4%)	
		AKEGGGSRGFRPPGALYY	1 (3%)	QHYGDSPPIT	1 (4%)	
		AKLRDGYNCSDY	1 (3%)	QKYNSAPWT	1 (4%)	
		AKVSYSNSGYYYYGMDV	1 (3%)	QQFNSYPLT	1 (4%)	
		ARAYCGDDCPVGYYYYGMDV	1 (3%)	QQFNSYPSLT	1 (4%)	
		ARDYYGSGPDY	1 (3%)	QQSYNIPRT	1 (4%)	
		AREYINSWYHYFDS	1 (3%)	QQSYSTPLT	1 (4%)	
		ARGFDGYKVGGH	1 (3%)	QQSYSTPPVT	1 (4%)	
		ARGFGNDTFFMVYGFDA	1 (3%)	QQSYSTPT	1 (4%)	
		ARGGNWKGDDY	1 (3%)	QQSYSVPLT	1 (4%)	
		ARGNRGEQFDSGGYYCFWFDP	1 (3%)	QQYGSSPLQGLT	1 (4%)	
		ARGTQGNNFGGDC	1 (3%)	QQYSGYWT	1 (4%)	
		ARSLDVGASMPFDY	1 (3%)	QQYYSTPLT	1 (4%)	
		GRGPYYYDSGTYFNAYIDN	1 (3%)	QQYYSTPRT	1 (4%)	
		TTDGALRPYQFVY	1 (3%)	QQYYSYPSTWT	1 (4%)	
BC		**IgG (n=33)**		**Igκ (n=35)**		
		ARILRVAGFDY	4 (12%)	QQYNNWPPWT	2 (6%)	
		ARMGGGYAGENAFDV	2 (6%)	QQYNNWPTWT	2 (6%)	
		AHSGWSTSVLEY	2 (6%)	QQYNNWPYT	2 (6%)	
		ARMVGSYGVQGQWLALYDS	2 (6%)	QQYYSYPPT	2 (6%)	
		ARFPMFRGVDY	2 (6%)	LQHNSYPYT	1 (3%)	
		AHRGVGGRHCC	1 (3%)	MQGTHWPPWT	1 (3%)	
		AQAWGIGGSGGTSLDL	1 (3%)	MQTTRDVS	1 (3%)	
		AHIKSLFTGSAGDYGMDV	1 (3%)	QHLNSYPR	1 (3%)	
		ARIVNFHWFLNYFDY	1 (3%)	QKYNNWPRGT	1 (3%)	
		ARITGTTGYFDY	1 (3%)	QLFGTSQYT	1 (3%)	
		ARMRPFSGNIGTYHLDC	1 (3%)	QQFNSYPPLT	1 (3%)	
		ARSGWSSSVLED	1 (3%)	QQFYSLPLT	1 (3%)	
		ARTLRREIGSYPFDF	1 (3%)	QQYDNLLMYT	1 (3%)	
		ARSPGDDYGDYFFDY	1 (3%)	QQYHKWPPVT	1 (3%)	
		AKALGRSGYGRFDY	1 (3%)	QQYNNWPPWFT	1 (3%)	
		AREAGDSDGRAFDI	1 (3%)	QQYNNWPPYT	1 (3%)	
		AKYGEFMIVVPHFDY	1 (3%)	QQYNNWPQT	1 (3%)	
		AKGTTNIDY	1 (3%)	QQYNNWPRT	1 (3%)	
		AKDVRGGTTDLPDY	1 (3%)	QQYNNWPSGT	1 (3%)	
		AKDIWRWAFDY	1 (3%)	QQYNSY	1 (3%)	
		ARDSYSSGWYFDY	1 (3%)	QQYNSYPWT	1 (3%)	
		AKGESGTMDV	1 (3%)	QQYNSYSYT	1 (3%)	
		ARIPGSYFTGRYYFDY	1 (3%)	QQYNTYWT	1 (3%)	
		ARDFGYSYHRPIHWFFDL	1 (3%)	QQYQTLPLT	1 (3%)	
		ARQDYGDYRLGY	1 (3%)	QQYSSSFPWT	1 (3%)	
		ARVAVGRFLEWDHPRAWYFDL	1 (3%)	QQYTADSSWT	1 (3%)	
				QQYYSTPRFT	1 (3%)	
				QQYYSTPWT	1 (3%)	
				QQYYTPLT	1 (3%)	
				QQYNNWPPLT	1 (3%)	
				QQYDNLFT	1 (3%)	
