# Supplementary material for: Expression of combinatorial immunoglobulins in macrophages in the tumor microenvironment
Source: PLoS One. 2018 Sep 21;13(9):e0204108. doi: 10.1371/journal.pone.0204108 (PMC6150476; doi:10.1371/journal.pone.0204108)
Supplement: S2 Table — (PDF) [file pone.0204108.s013.pdf]

Table S2: Shared IgG, IgM and Igκ CDR3 protein sequences among different cell fractions

	TAM-1		TAM-2	TAM-3	BC
	IgM	IgG	IgM	IgM	IgG
ARVPINYGILTGTDY			37	37	
ARIPGSYFTGRYYFDY	1	7			1

	TAM-2	TAM-3	BCI-1	BCI-2	BCI-3	BC
	Igκ					
QQYDNLPYT	5	10				
QQYDNLSALT	5	6				
QQYNSYSYT	1					1
QQYNTYPLT		19	1			
QQSYSTPRT		1	2			
QQYNSYPWT				1		1
QQSYSTPYT			2		3	
QQYYSTPLT			1		1	
QQSYSTPLT				1	1	

Several CDR3s were found to be shared among repertoires. Two Ig heavy chain CDR3s occurred in more than one repertoire. Several CDR3s were shared among the Igκ light chain repertoires.
