# Supplementary material for: Expression of combinatorial immunoglobulins in macrophages in the tumor microenvironment
Source: PLoS One. 2018 Sep 21;13(9):e0204108. doi: 10.1371/journal.pone.0204108 (PMC6150476; doi:10.1371/journal.pone.0204108)
Supplement: S3 Table — (PDF) [file pone.0204108.s014.pdf]

Table S3: List of all V(D)J combinations found in the sanger-sequenced cell fractions.

Sample	IgM		IgG		Igκ		Igλ	
TAM-1	**IgM (n=34)**		**IgG (n=37)**		**Igκ (n=15)**			
	VH2-70/D2-2/J4	26 (76%)	VH4-31/D3-3/J4	18 (49%)	VK1-5/J4	9 (60%)		
	VH5-51/D3-10/J3	5 (15%)	VH4-31/D3-3/J6	8 (22%)	VK3-20/J4	4 (27%)		
	VH3-23/D1-14/J4	2 (6%)	VH4-4/D1-26/J4	7 (19%)	VK1-16/J1	2 (13%)		
	VH4-4/D1-26/J4	1 (3%)	VH4-4/D2-15 inv/J3	3 (8%)				
			VH4-31/D3-16/J4	1 (3%)				
TAM-2	**IgM (n=39)**		**IgG (n=34)**		**Igκ (n=28)**		**Igλ (n=36)**	
	VH3-21/D3-9/J4	39 (100%)	VH4-61/D2-2/J3	20 (59%)	VK1-33/J4	6 (21%)	VL7-46/J3	29 (81%)
			VH1-18/D3-3/J6	10 (29%)	VK1-5/J2	6 (21%)	VL3-21/J3	5 (14%)
			VH1-69/D3-10/J4	2 (6%)	VK1-33/J2	5 (18%)	VL3-1/J3	2 (6%)
			VH3-30/D3-10/J4	2 (6%)	VK3-20/J2	4 (14%)		
					VK3-20/J1	3 (11%)		
					VK3-15/J4	2 (7%)		
					VK1-5/J3	1 (4%)		
					VK2-28/J5	1 (4%)		
TAM-3	**IgM (n=50)**				**Igκ (n=36)**			
	VH3-21/D3-9/J4	39 (78%)			VK1-5/J4	19 (53%)		
	VH3-74/-/J6	11 (22%)			VK1-33/J4	6 (17%)		
					VK1-33/J2	5 (14%)		
					VK1-5/J2	5 (14%)		
					VK1-39/J2	1 (3%)		
BCI-1			**IgG (n=39)**		**Igκ (n=35)**			
			VH1-69/D3-3/J3	3 (8%)	VK3-20/J4	5 (14%)		
			VH1-18/D4-11/J4	2 (5%)	VK1-39/J1	4 (11%)		
			VH7-4-1/D5-24/J4	2 (5%)	VK1-33/J2	3 (9%)		
			VH1-8/D2-21/J4	2 (5%)	VK1-39/J2	3 (9%)		
			VH7-4-1/D3-22/J5	2 (5%)	VK1-39/J4	3 (9%)		
			VH5-51/D1-26/J4	2 (5%)	VK1-5/J1	2 (6%)		
			VH1-2/D2-21/J4	1 (3%)	VK1-33/J4	2 (6%)		
			VH1-69/D6-19/J5	1 (3%)	VK4-1/J4	2 (6%)		
			VH3-11/D3-9/J4	1 (3%)	VK1-5/J4	1 (3%)		
			VH3-21/D2-21/J6	1 (3%)	VK1-8/J4	1 (3%)		
			VH3-30/D6-19/J3	1 (3%)	VK1-9/J2	1 (3%)		
			VH3-30/D3-22/J4	1 (3%)	VK1-12/J3	1 (3%)		
			VH3-30/D2-21/J6	1 (3%)	VK1-33/J1	1 (3%)		
			VH3-30/D5-12/J4	1 (3%)	VK3-11/J2	1 (3%)		
			VH4-61/D3-10/J3	1 (3%)	VK3-20/J1	1 (3%)		
			VH7-4-1/D1-7/J5	1 (3%)	VK3-20/J5	1 (3%)		
			VH1-2/D2-8/J4	1 (3%)	VK4-1/J2	1 (3%)		
			VH1-8/D3-10/J4	1 (3%)	VK5-2/J1	1 (3%)		
			VH1-46/D5-24/J4	1 (3%)	VK5-2/J2	1 (3%)		
			VH3-7/D3-10/J4	1 (3%)				
			VH3-9/D2-2/J6	1 (3%)				
			VH3-21/D3-9/J4	1 (3%)				
			VH3-23/D3-16/J3	1 (3%)				
			VH3-23/D5-24/J4	1 (3%)				
			VH3-30/D6-19/J4	1 (3%)				
			VH3-30/D2-21/J4	1 (3%)				
			VH3-33/D5-12/J3	1 (3%)				
			VH3-74/D6-19/J1	1 (3%)				
			VH3-74/D4-23/J4	1 (3%)				
			VH4-59/D4-17/J4	1 (3%)				
			VH5-51/D1-7/J5	1 (3%)				
			VH3-23/D2-21/J6	1 (3%)				
BCI-2			**IgG (n=18)**		**Igκ (n=25)**			
			VH4-39/D1-26/J6	5 (28%)	VK1-5/J1	4 (16%)		
			VH4-31/D6-13/J4	3 (17%)	VK1-39/J1	3 (12%)		
			VH4-39/D2-15/J2	2 (11%)	VK1-5/J2	2 (8%)		
			VH4-39/D6-6/J5	2 (11%)	VK1-39/J4	2 (8%)		
			VH1-8/D2-15/J5	1 (6%)	VK3-20/J4	2 (8%)		
			VH1-18/D1-20/J5	1 (6%)	VK4-1/J1	2 (8%)		
			VH3-15/D6-19/J4	1 (6%)	VK1-6/J4	1 (4%)		
			VH4-31/D3-22/J5	1 (6%)	VK1-9/J4	1 (4%)		

		VH4-39/D5-24/J5	1 (6%)	VK1-12/J4	1 (4%)	
		VH4-61/D6-19/J4	1 (6%)	VK1-16/J1	1 (4%)	
				VK1-33/J3	1 (4%)	
				VK1-33/J4	1 (4%)	
				VK1-39/J2	1 (4%)	
				VK1-39/J5	1 (4%)	
				VK3-20/J1	1 (4%)	
				VK3-20/J2	1 (4%)	
**BCI-3**		**IgG (n=32)**		**Igκ (n=26)**		
		VH3-33/D2-15/J4	4 (13%)	VK1-5/J4	3 (12%)	
		VH3-9/D7-27/J6	3 (9%)	VK1-39/J2	3 (12%)	
		VH4-31/D4-17/J4	3 (9%)	VK1-39/J4	3 (12%)	
		VH4-31/D6-19/J3	2 (6%)	VK1D-13/J4	2 (8%)	
		VH3-11/D3-16/J5	2 (6%)	VK1-33/J5	2 (8%)	
		VH4-59/D2-2/J4	2 (6%)	VK1D-43/J1	1 (4%)	
		VH3-11/D6-13/J6	1 (3%)	VK1-6/J1	1 (4%)	
		VH3-30/D3-16/J4	1 (3%)	VK1-8/J1	1 (4%)	
		VH4-4/D5-18/J4	1 (3%)	VK1-8/J2	1 (4%)	
		VH4-31/D2-21/J6	1 (3%)	VK1-9/J2	1 (4%)	
		VH4-31/D3-9/J5	1 (3%)	VK1-17/J1	1 (4%)	
		VH1-2/D5-18/J5	1 (3%)	VK1-17/J4	1 (4%)	
		VH1-69/D3-10/J4	1 (3%)	VK1-27/J1	1 (4%)	
		VH3-15/D5-24/J4	1 (3%)	VK1-39/J1	1 (4%)	
		VH3-23/D2-15/J4	1 (3%)	VK1-39/J5	1 (4%)	
		VH3-33/D1-26/J4	1 (3%)	VK2-30/J1	1 (4%)	
		VH3-53/D6-13/J4	1 (3%)	VK3-20/J4	1 (4%)	
		VH3-48/D3-10/J4	1 (3%)	VK3-20/J5	1 (4%)	
		VH4-31/D1-1/J4	1 (3%)			
		VH4-31/D3-22/J5	1 (3%)			
		VH4-61/D5-24/J5	1 (3%)			
		VH4-28/D5-24/J4	1 (3%)			
**BC**		**IgG (n=33)**		**Igκ (n=35)**		
		VH2-26/D6-19/J4	4 (12%)	VK3-15/J1	6 (17%)	
		VH2-70/D3-10/J4	3 (9%)	VK1-5/J1	4 (11%)	
		VH2-26/D5-24/J3	2 (6%)	VK3-15/J2	4 (11%)	
		VH2-26/D2-8/J4	2 (6%)	VK1-5/J2	2 (6%)	
		VH2-70/D6-19/J4	2 (6%)	VK1-5/J4	2 (6%)	
		VH2-5/D1-26/J4	1 (3%)	VK1-9/J4	2 (6%)	
		VH2-5/D3-16/J4	1 (3%)	VK4-1/J1	2 (6%)	
		VH2-5/D3-22/J6	1 (3%)	VK4-1/J4	2 (6%)	
		VH2-26/D3-9/J4	1 (3%)	VK1-9/J1	1 (3%)	
		VH2-70/D1-7/J4	1 (3%)	VK1D-17/J2	1 (3%)	
		VH2-70/D2-2/J4	1 (3%)	VK1-33/J4	1 (3%)	
		VH2-70/D1-26/J4	1 (3%)	VK1-37/J2	1 (3%)	
		VH2-70/D4-17/J4	1 (3%)	VK1-39/J4	1 (3%)	
		VH3-15/D5-24/J4	1 (3%)	VK2-24/J2	1 (3%)	
		VH3-21/D2-21/J3	1 (3%)	VK2-28/J4	1 (3%)	
		VH3-23/D3-22/J4	1 (3%)	VK2-30/J1	1 (3%)	
		VH3-23/D2-2 inv/J4	1 (3%)	VK3-15/J3	1 (3%)	
		VH3-23/D6-19 inv./J4	1 (3%)	VK3-20/J2	1 (3%)	
		VH3-23/D3-3/J4	1 (3%)	VK4-1/J3	1 (3%)	
		VH3-30/D6-19/J4	1 (3%)			
		VH3-30/D3-10/J6	1 (3%)			
		VH4-4/D1-26/J4	1 (3%)			
		VH4-31/D3-3/J2	1 (3%)			
		VH4-59/D5-18/J2	1 (3%)			
		VH5-51/D4-17/J4	1 (3%)			
