# Supplementary material for: Expression of combinatorial immunoglobulins in macrophages in the tumor microenvironment
Source: PLoS One. 2018 Sep 21;13(9):e0204108. doi: 10.1371/journal.pone.0204108 (PMC6150476; doi:10.1371/journal.pone.0204108)
Supplement: S4 Table — (PDF) [file pone.0204108.s015.pdf]

Table S4: Shared V(D)J recombinations among different repertoires and cell types. Only functional recombinations are shown.

	TAM-1		TAM-2		TAM-3	BCI-1	BCI-2	BCI-3	BC
	IgM	IgG	IgM	IgG	IgM	IgG	IgG	IgG	IgG
V_H1-69/D3-10/J_H4				2				1	
V_H2-70/D2-2/J_H4	26								1
V_H3-15/D5-24/J_H4								1	1
V_H3-21/D3-9/J_H4			39		39	1			
V_H3-30/D6-19/J_H4						1			1
V_H4-31/D3-22/J_H5							1	1	
V_H4-4/D1-26/J_H4	1	7							1

	TAM-1	TAM-2	TAM-3	BCI-1	BCI-2	BCI-3	BC
	Igκ						
Vκ1-16/Jκ1	2				1		
Vκ1-33/Jκ2		5	5	3			
Vκ1-33/Jκ4		6	6	2	1		1
Vκ1-39/Jκ1				4	3	1	
Vκ1-39/Jκ2			1	3	1	3	
Vκ1-39/Jκ4				3	2	3	1
Vκ1-39/Jκ5					1	1	
Vκ1-5/Jκ1				2	4		4
Vκ1-5/Jκ2		6	5		2		2
Vκ1-5/Jκ4	9		19	1		3	2
Vκ1-9/Jκ2				1		1	
Vκ1-9/Jκ4					1		2
Vκ2-30/Jκ1				1			1
Vκ3-20/Jκ1		3		1	1		
Vκ3-20/Jκ2		4			1		1
Vκ3-20/Jκ4	4			5	2	1	
Vκ3-20/Jκ5				1		1	
Vκ4-1/Jκ1					2		2
Vκ4-1/Jκ4				2			2
