# Supplementary material for: Expression of combinatorial immunoglobulins in macrophages in the tumor microenvironment
Source: PLoS One. 2018 Sep 21;13(9):e0204108. doi: 10.1371/journal.pone.0204108 (PMC6150476; doi:10.1371/journal.pone.0204108)
Supplement: S5 Table — (PDF) [file pone.0204108.s016.pdf]

Table S5: CDR3 sequences and the underlying VJ recombinations found in the NGS-sequenced samples TAM-4 and TAM-5.

	CDR3	IGHV	IGHJ	n
TAM-4	VKDVLTVSGAGWGDC	hIGHV3-23	hIGHJ4	712
	ARRYFDTRGHPLDF	hIGHV3-7	hIGHJ4	281
	ARRYFDTRGHPLDF	hIGHV3-66	hIGHJ4	220
	ARDYQQLGRGEDA	hIGHV4-61	hIGHJ6	147
	ARRYFDTRGHPLDF	hIGHV3-33	hIGHJ4	106
	ARDGGSGSYNWFDP	hIGHV3-7	hIGHJ5	95
	ARDYQQLGRGEDV	hIGHV4-61	hIGHJ6	83
	ARVASGSSTVQWDY	hIGHV4-61	hIGHJ4	80
	ARDGGSGSYNWFDP	hIGHV3-66	hIGHJ5	69
	ARDRDFWSGSVNYFDY	hIGHV1-18	hIGHJ4	48
	ARRYFDTRGHPLDF	hIGHV3-74	hIGHJ4	41
	ARDGGSGSYNWFDP	hIGHV3-33	hIGHJ5	33
	ARDGGSGSYNWFDP	hIGHV3-74	hIGHJ5	16
	ARDRDFWSGSVNYFDY	hIGHV1-46	hIGHJ4	8
	ARRYFDTRGHPLDF	hIGHV3-64	hIGHJ4	8
	AREWGSGLTLSWSFDL	hIGHV3-66	hIGHJ2	8
	AKDISPHYYDSSDLQH	hIGHV3-9	hIGHJ1	8
	ARDRDFWSGSVNYFDY	hIGHV1-69	hIGHJ4	7
	VKDVLAVSGAGWGDC	hIGHV3-23	hIGHJ4	7
	AKPRAGGDYAFDI	hIGHV3-23	hIGHJ3	7
	AREWGSGLTLSWSFDL	hIGHV3-7	hIGHJ2	7
	AKEDTVRISHWYFDL	hIGHV3-30-3	hIGHJ2	6
	VKDVLTVSGAGWGDC	hIGHV3-64	hIGHJ4	5
	ARLPEDTGRALDY	hIGHV3-11	hIGHJ4	4
	AKDPDSSGWINWYFDL	hIGHV3-23	hIGHJ2	4
	AKTNTLMTIWGSYDY	hIGHV3-30-3	hIGHJ4	4
	VKDVPTVSGAGWGDC	hIGHV3-23	hIGHJ4	3
	VKDVLTVSGAGWGDR	hIGHV3-23	hIGHJ5	3
	AREWGSGLTLSWSFDL	hIGHV3-33	hIGHJ2	3
	ARDCSPDLGDMYYDALDI	hIGHV3-48	hIGHJ3	3
	ARRYFDTRGHPLGF	hIGHV3-66	hIGHJ5	3
	AKTNTLMTIWGSYDY	hIGHV3-66	hIGHJ4	3
	TTILRYFDWLLHFDY	hIGHV3-15	hIGHJ4	2
	VKGVLTVSGAGWGDC	hIGHV3-23	hIGHJ4	2
	VKDVLTVSGARWGDC	hIGHV3-23	hIGHJ4	2
	VKDVLTVSGAGWGDC	hIGHV3-23	hIGHJ5	2
	VKDALTVSGAGWGDC	hIGHV3-23	hIGHJ4	2
	AKGRNFGVVIAFDY	hIGHV3-23	hIGHJ4	2
	AKDRGGGQWLDDALDY	hIGHV3-23	hIGHJ4	2
	ARRYFNTRGHPLDF	hIGHV3-33	hIGHJ4	2
	ARRYFDTRGHPLDF	hIGHV3-53	hIGHJ4	2
	ARDCSPDLGDMYYDALDI	hIGHV3-53	hIGHJ3	2
	ARDRDFWSGSVNYFDY	hIGHV3-64	hIGHJ4	2
	ARRYLDTRGHPLDF	hIGHV3-66	hIGHJ4	2
	AKEDTVRISHWYFDL	hIGHV3-66	hIGHJ2	2
	ARRYLDTRGHPLDF	hIGHV3-7	hIGHJ4	2
	ARRYFGTRGHPLDF	hIGHV3-7	hIGHJ4	2
	ARDYQQPGRGEDA	hIGHV4-61	hIGHJ6	2
	ARAGDYGEYYFDY	hIGHV4-61	hIGHJ4	2
	ATTSSLYYYGMDV	hIGHV6-1	hIGHJ6	2

	CDR3	IGHV	IGHJ	n
TAM-5	ARRYYYASIGHYSYDL	hIGHV5-51	hIGHJ5	2982
	ARRYYYASIGHYSYDL	hIGHV5-a	hIGHJ5	875
	AIYPWYYCSINTCPLAFDP	hIGHV3-48	hIGHJ5	46
	ARRYYYASIGHYSYGL	hIGHV5-51	hIGHJ5	18
	ARRYYYASIGHYSYVL	hIGHV5-51	hIGHJ5	17
	ARRYYYASIGHYPYDL	hIGHV5-51	hIGHJ5	16
	AIYPWYYCSINTCPLAFDP	hIGHV3-66	hIGHJ5	13
	AGRYYYASIGHYSYDL	hIGHV5-a	hIGHJ5	12
	ARRYCYASIGHYSYDL	hIGHV5-51	hIGHJ5	11
	ARRYYYAGIGHYSYDL	hIGHV5-51	hIGHJ5	9
	ARRCYYASIGHYSYDL	hIGHV5-51	hIGHJ5	7
	ARRYYYASIGHYSYGL	hIGHV5-a	hIGHJ5	7
	ARRHYYASIGHYSYDL	hIGHV5-51	hIGHJ5	6
	ARRYYHASIGHYSYDL	hIGHV5-51	hIGHJ5	6
	ARHYYYASIGHYSYDL	hIGHV5-51	hIGHJ5	5
	ARRYHYASIGHYSYDL	hIGHV5-51	hIGHJ5	5
	ARRYYYASTGHYSYDL	hIGHV5-51	hIGHJ5	5
	ARRYYYAGIGHYSYDL	hIGHV5-a	hIGHJ5	4
	ARRYYYASIGHYSHDL	hIGHV5-51	hIGHJ5	4
	ARRYYYARIGHYSYDL	hIGHV5-51	hIGHJ5	3
	ARRYYYASIGHYPYDL	hIGHV5-a	hIGHJ5	3
	ARRYYYASIGHYSCDL	hIGHV5-51	hIGHJ5	3
	ARRYYYASIGRYSYDL	hIGHV5-51	hIGHJ5	3
	ARRYYYASIGYYSYDL	hIGHV5-a	hIGHJ5	3
	ARRYYYASVGHYSYDL	hIGHV5-51	hIGHJ5	3
	ARRYYYVSIGHYSYDL	hIGHV5-51	hIGHJ5	3
	AIYPWYYCSINTCPLAFDP	hIGHV3-64	hIGHJ5	2
	AKRYYYASIGHYSYDL	hIGHV5-a	hIGHJ5	2
	ARRYYYASIGHYSHDL	hIGHV5-a	hIGHJ5	2
	ARRYYYASIGHYSYNL	hIGHV5-51	hIGHJ5	2
	ARRYYYASIGHYSYYL	hIGHV5-51	hIGHJ5	2
	ARRYYYASIGYYSYDL	hIGHV5-51	hIGHJ5	2
	ARRYYYASISHYSYDL	hIGHV5-51	hIGHJ5	2
	ARRYYYASNGHYSYDL	hIGHV5-51	hIGHJ5	2
	ARRYYYVSIGHYSYDL	hIGHV5-a	hIGHJ5	2
	TRRYYYASIGHYSYDL	hIGHV5-51	hIGHJ5	2
	VRRYYYASIGHYSYDL	hIGHV5-51	hIGHJ5	2
