# Supplementary material for: Expression of combinatorial immunoglobulins in macrophages in the tumor microenvironment
Source: PLoS One. 2018 Sep 21;13(9):e0204108. doi: 10.1371/journal.pone.0204108 (PMC6150476; doi:10.1371/journal.pone.0204108)
Supplement: S6 Table — (PDF) [file pone.0204108.s017.pdf]

Table S6: number of detected heavy and light chain sequences in single TAM isolated from different tumor samples

Patient	TAM isolated from	number of analyzed cells	number of detected heavy chains	number of detected light chains
**TAM-6**	liver ca	25	7	0
**TAM-7**	colon ca	17	0	0
**TAM-8**	lymphoma	17	0	0
**TAM-9**	colon ca	29	1	2
**TAM-10**	glioblastoma	14	0	0
